# Genome-wide characterization of *LEA* gene family reveals a positive role of *BnaA.LEA6.a* in freezing tolerance in rapeseed (*Brassica napus* L*.*)

**DOI:** 10.1186/s12870-024-05111-7

**Published:** 2024-05-21

**Authors:** Weiping Wang, Yan Liu, Yu Kang, Wei Liu, Shun Li, Zhonghua Wang, Xiaoyan Xia, Xiaoyu Chen, Lunwen Qian, Xinghua Xiong, Zhongsong Liu, Chunyun Guan, Xin He

**Affiliations:** College of Agronomy, Hunan Agricultural University, Changsha, 410128 Hunan China

**Keywords:** *Brassica napus*, LEA (Late embryogenesis abundant), Freezing stress, Abiotic stress

## Abstract

**Background:**

Freezing stress is one of the major abiotic stresses that causes extensive damage to plants. LEA (Late embryogenesis abundant) proteins play a crucial role in plant growth, development, and abiotic stress. However, there is limited research on the function of *LEA* genes in low-temperature stress in *Brassica napus* (rapeseed).

**Results:**

Total 306 potential *LEA* genes were identified in *B. rapa* (79)*, B. oleracea* (79) *and B. napus* (148) and divided into eight subgroups. *LEA* genes of the same subgroup had similar gene structures and predicted subcellular locations. *Cis*-regulatory elements analysis showed that the promoters of *BnaLEA* genes rich in *cis*-regulatory elements related to various abiotic stresses. Additionally, RNA-seq and real-time PCR results indicated that the majority of *BnaLEA* family members were highly expressed in senescent tissues of rapeseed, especially during late stages of seed maturation, and most *BnaLEA* genes can be induced by salt and osmotic stress. Interestingly, the *BnaA.LEA6.a* and *BnaC.LEA6.a* genes were highly expressed across different vegetative and reproductive organs during different development stages, and showed strong responses to salt, osmotic, and cold stress, particularly freezing stress. Further analysis showed that overexpression of *BnaA.LEA6.a* increased the freezing tolerance in rapeseed, as evidenced by lower relative electrical leakage and higher survival rates compared to the wild-type (WT) under freezing treatment.

**Conclusion:**

This study is of great significance for understanding the functions of *BnaLEA* genes in freezing tolerance in rapeseed and offers an ideal candidate gene (*BnaA.LEA6.a*) for molecular breeding of freezing-tolerant rapeseed cultivars.

**Supplementary Information:**

The online version contains supplementary material available at 10.1186/s12870-024-05111-7.

## Introduction

Plants, as sessile organisms, are susceptible to abiotic stresses throughout their growth and development. Due to global climate change, the frequent occurrence of extreme weather events has increased the risk of low-temperature stress in plants, which in turn severely affects crop yields [1, 2]. Plant cell membranes are most susceptible to cold damage [3–5]. Chilling temperatures (above 0 ℃) have impact on the fluidity of cell membranes and thus hindering plant growth and development, while freezing temperature (below 0 ℃) result in the disruption of cell membranes, ultimately leading cell death [6]. Plants have evolved various adaptations at the molecular, cellular, and physiological level to cope with different growth conditions, one such adaption involves the expression of a specific class of proteins known as late embryogenesis abundant (LEA) proteins [7–10].

LEA proteins are a class of proteins characterized by their high hydrophilicity, thermal stability, and intrinsic disorder. Most LEA proteins contain hydrophilic amino acids such as glycine, lysine, and histidine [11]. Based on sequence similarity and protein family domains (Pfam), LEA proteins can be divided into eight subgroups: LEA_1, LEA_2, LEA_3, LEA_4, LEA_5, LEA_6, DHN (Dehydrin), and SMP (Seed Maturation Protein) [12]. LEA proteins were first discovered in the late stages of cotton seed maturation and have since been found in roots, stems, leaves, and flowers of plants [13, 14]. It has been shown that LEA proteins can maintain basic cellular metabolism by adsorbing water molecules under dehydration conditions and stabilize membranes by binding to them [15, 16]. Additionally, LEA proteins play important roles in maintaining the stability of the cellular matrix in a glassy state, ionic binding, molecular protection, and preservation of enzyme activity and double-stranded DNA binding [17–19]. These characteristics make LEA proteins play important role in protecting plants from abiotic stress conditions. Overexpression of a cucumber Y3SK2 type DHN gene *CsLEA11* in *E. coli* enhanced cell viability and conferred heat and cold tolerance [20]. Overexpression of maize *ZmLEA3* enabled tobacco, yeast, and *E. coli* to tolerate low-temperature stress [21]. Tomato strains expressing *SiLEA4* had significantly greater freezing resistance, due to a significant increase in the antioxidize activities and proline content [22]. Both *LEA3* from rapeseed and *Arabidopsis* primarily improve drought tolerance and oil content by enhancing photosynthetic efficiency and reducing reactive oxygen species (ROS) accumulation [23]. Weighted gene co-expression network analysis revealed that late embryogenesis abundant (LEA) protein genes contribute to water absorption and transportation during seed germination under low-temperature stress [24]. Overexpression of the *BcLEA73* gene in *Arabidopsis* has been found to significantly enhance their tolerance to salt, drought, osmotic stress, and low temperature [25].

Rapeseed (*Brassica napus*: genome AnAnCnCn) is an important oil-seed crop, which was formed ~ 7500 years ago by allopolyploid between the ancestor of *B. rape* (genome ArAr) and *B. oleracea* (genome CoCo) [26, 27]. The growth and development of rapeseed are severely impacted by environmental conditions. Although there were some studies about the functions of *LEA* genes in salt and drought tolerance, seed oil content in rapeseed, the research about their functions in freezing tolerance in rapeseed remains limited. In this study, 306 putative *LEA* genes were identified in *B. rapa*, *B. oleracea* and *B. napus*, and their structural features, chromosome localization, evolutionary relationship, promoter sequence, and expression patterns in various tissues and under different abiotic stresses were evaluated. Additionally, we confirmed that overexpression of the *BnaA.LEA6.a* gene enhances the freezing tolerance in rapeseed seedlings. This study will provide valuable information for deciphering the biological functions of *BnaLEA* genes in the response of freezing stress in rapeseed.

## Materials and methods

### Identification of *LEA *genes in *B. rapa*, *B. oleracea *and *B. napus*

The protein, CDS, and DNA sequences of *Arabidopsis*, *B. rapa* and *B. oleracea* LEAs were downloaded from Ensembl genomes (http://ensemblgenomes.org/). Whole-gene data of *Brassica napus* was downloaded from *Brassica napus* pan-genome information resource (http://cbi.hzau.edu.cn/bnapus/) [28]. Hidden Mark Model profiles of LEA proteins with accession numbers PF03760 (LEA_1), PF03168 (LEA_2), PF03242 (LEA_3), PF02987 (LEA_4), PF00477 (LEA_5), PF10714 (LEA_6), PF00257 (DHN), and PF04927 (SMP) downloaded from the Pfam database (https://pfam.xfam.org/), were used to HMM search against the local genome database of *B. napus* using TBtoolsv.120 [29]. Fifty-one AtLEA proteins were used as query sequences for the search of *B. napus, B. rapa* and *B. oleracea* LEA proteins by BLASTP (E-value < 1e^−5^) in TBtoolsv.120 [29]. All members were verified for the presence of LEA repeats using Conserved Domain (CD)-search in NCBI (https://www.ncbi.nlm.nih.gov/Structure/). The subcellular localizations of LEAs were predicted using WOLF PSORT (https://wolfpsort.hgc.jp/). The molecular weight (kDa), theoretical isoelectric point (pI) and grand average of hydropathy (GRAVY) of LEAs were predicted by EXPASY (https://web.expasy.org/protparam/) [30]. The TBtoolsv1.120 [29] was used to analyze the *LEAs* gene structure. The conserved motifs were analyzed with MEME program (http://meme.nbcr.net/meme/cgi-bin/meme/cgi) [31].

### Chromosomes position and collinearity analysis of *LEAs*

The chromosomal distribution of *LEA* genes was determined by using TBtools v1.120 based on the GTF file. Using the One Step MCScanX and Circos functions in TBtools v1.120, the *LEA* genes in *Arabidopsis*, *B. rapa*, *B. oleracea*, and *B. napus* were analyzed in collinearity based on genome files and GTF files. Gene duplications were identified by collinearity and phylogenetic analysis.

### Phylogenetic analysis of the LEA proteins

The sequence of LEA proteins was aligned using Muscle, and a phylogenetic tree was constructed using the Maximum-likelihood (ML) phylogenetic method with 1000 bootstrap replicates in TBtoolsv.120 [29].

### Prediction of *cis*-regulatory elements in promoter sequences of* BnaLEA* genes 

The 2 kb promotor region of each *BnaLEA* gene was submitted to the PlantCARE (http://bioinformatics.psb.ugent.be/webtools/plantcare/html/) promoter analysis tool to identify potential *cis*-regulatory elements.

### Expression analysis of *BnaLEA* genes

To analyze the expression pattern of *BnaLEA* genes in various tissues as well as under phytohormonal and abiotic stresses of *B. napus*, the publicly available RNA-seq dataset of ZS11 (semi-winter type *B. napus* cv. Zhongshuang 11) were retrieved from BnIR (https://yanglab.hzau.edu.cn/BnIR) [28]. Gene expression levels were calculated using the TPM method (transcripts per million), and the expression values of the *BnaLEA* gene were shown in the Table S3. The log_2_(TPM + 1) value of the *BnaLEA* gene was used to generate heat map by TBtoolsv1.120 [29]. qRT-PCR analysis of *BnaA.LEA6.a* was performed as described previously [32–34]. The primers used in this study were designed in NCBI (https://www.ncbi.nlm.nih.gov/tools/primer-blast/) and listed in Table S4.

### Vector construction and plant phenotype assay

In this paper, the semi-winter rapeseed variety Zhongshuang 6 was obtained commercially from Wuhan Towin Biotechnology Co., Ltd., Wuhan, China. We constructed and transferred the 35S overexpression vector of *BnaA.LEA6.a* to Zhongshuang 6. The relative expression level of *BnaA.LEA6.a* gene in *BnaA.LEA6.a*-overexpression lines and wild-type (WT) were analyzed by qRT-PCR. Two *BnaA.LEA6.a* -overexpression lines (OE3, and OE4) were selected for subsequent experiments. *BnaA.LEA6.a* -overexpression lines and WT were seeded in nutrient soil and placed in an artificial climate chamber for growth. The growth conditions were as described previously [35]. The 30-day-old seedlings of WT and *BnaA.LEA6.a* -overexpression lines were subjected to freezing treatments. The freezing treatment was carried out by reducing the temperature from 20 ℃ to 0 ℃, and then reduced to -3.5 ℃ in a gradient of 1 ℃ per hour, with the -3.5 °C lasting for 2 h, followed by recovery at 20 ℃ for 7 days. The phenotypic change of *B. napus* were photographed, and the survival rate was counted. The relative electrical conductivity contents were detected by the method according to provious study [36]. Three replicates were performed for each sample.

### Statistical analysis

All results in this study were performed in more than three replicates. Data were expressed as mean of triplicate values, and the error represented the SEM (Standard Error of Mean). Statistical analysis and plotting were performed using GraphPad Prism8 (V8.4.3, GraphPad, Changchun, China). The statistical significance of difference was confirmed by one-way ANOVA.

## Results

### Genome-wide identification of LEAs in *B. rapa*, *B. oleracea *and *B. napus*

A total of 306 LEA proteins were identified in *B. rape* (79)*, B. oleracea* (79) *and B. napus* (148)*.* based on the BLASTP results of AtLEAs protein sequence and Hidden Mark Model (Table S1). Based on their conserved domain structures, the LEA proteins were classified into eight families. Most proteins in the same subgroup had similar parameters. The 306 LEA proteins encoded 57–298 amino acids (AA) with the CDS ranging from 174 bp (*BnaA.LEA1.d*) to 897 bp *(BnaC.LEA4-4.a*). The predicted molecular weights (Mw) of 87.25% (267/306) LEA proteins ranged from 6.61 kDa (BnaA.LEA1.d) to 33.66 kDa (BnaC.LEA4-4.a). All members of LEA_2 (18/18) and the majority of LEA_4 (69/133), LEA_5 (9/10), LEA_6 (13/14), DHN (35/53), and SMP (29/31) subgroups had an isoelectric point (pI) < 7. Most members of LEA_1 (17/18) and LEA_3 (25/28) subgroups had a pI > 7. The grand average of hydropathicity (GRAVY) of all LEAs calculated on the website ranges from -1.893 to 0.312 and GRAVY values of 287 LEA proteins (93.79%) were below 0. Subcellular localization predictions for these proteins suggest that most LEA proteins may be in the nucleus (109), with a few in the mitochondria (42), chloroplasts (78), cytoplasm (33), or extracellular space (32).

### Phylogenetic analysis

**Fig. 1:**
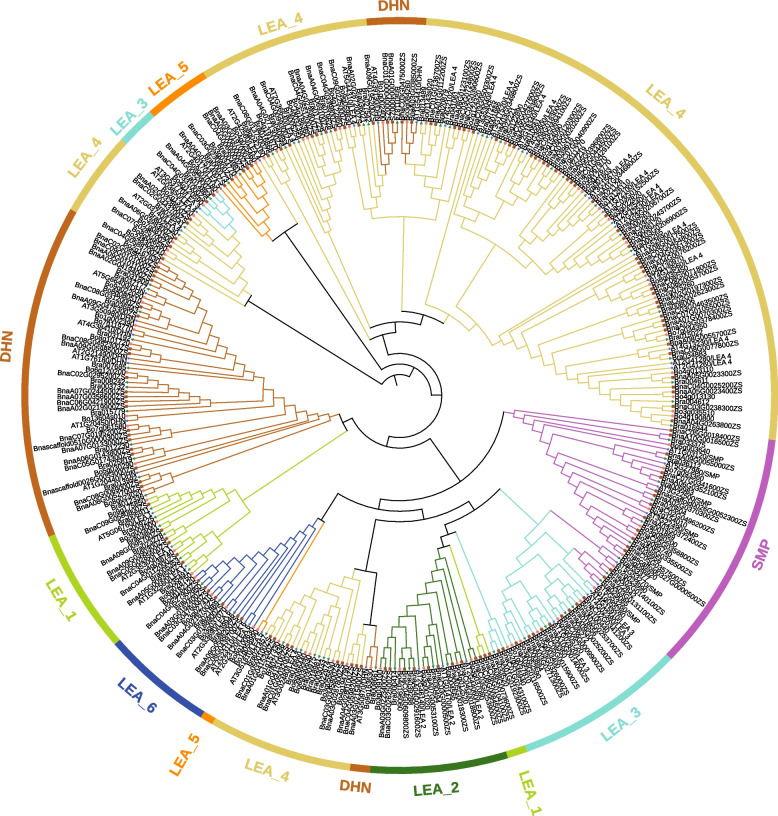
Phylogenetic tree of 357 LEA proteins from *B. rapa* (79), *B. oleracea* (79), *B. napus* (148) and *Arabidopsis* (51). A Maximum Likelihood phylogenetic tree was generated with full-length LEA protein sequences (1000 bootstrap replicates). The eight resulting groups (LEA_1, LEA_2, LEA_3, LEA_4, LEA_5, LEA_6, SMP and DHN) are labeled by different colors

A phylogenetic tree was generated using the sequences of 357 LEA proteins from *Arabidopsis* (51), *B. napus* (148), *B. rapa* (79), and *B. oleracea* (79) (Fig. 1). The LEA proteins were classified into eight groups, with the LEA_4 subgroup being the largest with 133 members, mainly clustered in three branches. Members of the LEA_1, LEA_3, LEA_5, DHN, and SMP subgroups were clustered in two branches, while LEA_2 and LEA_6 members form a separate branch. All 51 *AtLEAs* have orthologous in *B. rapa*, *B. oleracea* and *B. napus*. Based on the distribution of orthologous genes in the Ar (*B. rapa*), Co (*B. oleracea*), and the An- and Cn-subgenomes of *B. napus*, 36 pairs of Ar-Co-An-Cn genes were identified. Additionally, the *AT1G02820/LEA_3* gene underwent LF (Least Fractioned subgenome), MF1 (More Fractioned subgenomes 1), and MF2 (More Fractioned subgenomes 2) duplication in *B. rapa*, *B. oleracea* and *B. napus* [37].

### Motif analysis and gene structure analysis of LEAs

To explore the conserved LEAs motifs, four motifs were identified using MEME (Fig. S1). The result showed that the distribution patterns of these four motifs were similar in LEA members from same subgroup, such as LEA_1 (motif-1–3-2–4), LEA_2 (motif-1–2-4–3), LEA_3 (motif-1–4-2–1),LEA_5 (motif-4–1-2–3), DHN (motif-4–3-1–2), and SMP (motif- motif-4–3-1–2), whereas in other subgroups, such as LEA_2, LEA_4, and LEA_6, although the distribution patterns of motifs varied among the different members, the distribution of AtLEAs and their orthologous BnaLEAs were similar.

To investigate the structural characteristics of *LEA* genes, the exon–intron structure (Fig. S2 and Table S1) of *LEA* genes were analyzed. The results indicated that members of the same subgroups have similar gene structures. All LEA_6 subgroup genes had only one exon (except *BnaC.LEA6.d* with 2 exons), all members of LEA_5 (10) subgroup had 2 exons and the majority of LEA_1 (19/20), LEA_2 (16/17), LEA_3 (26/28), LEA_4 (128/133), DHN (29/31), and all SMP (53) subgroup members have 1 to 3 exons.

### Synteny and chromosomal location analysis in *Arabidopsis*, *B. rapa*, *B. oleracea* and *B. napus*

To analyze the collinear of *LEA* genes in *Arabidopsis*, *B. rapa*, *B. oleracea*, and *B. napus*, collinear gene pairs were obtained using TBtools v1.120. As shown in Fig. S3, the Ar-subgenomes in *B. rapa* were collinear with the An-subgenomes in *B. napus*, while the Co-subgenomes in *B. oleracea* were largely collinear with the Cn-subgenomes in *B. napus*. There were 66 *LEA* genes both in *B*. *rapa* and *B. oleracea* found collinear pairs in *B. napus*, and both *B. rapa* and *B. oleracea* had 13 genes that were not collinear in *B. napus*. 83.54% (66/79) of the Ar-subgenomes and Co-subgenomes were collinear.

To understand the distribution of *LEA* genes on the chromosomes of *B. rapa*, *B. oleracea*, and *B. napus*, chromosomal localization analysis was conducted using TBtoolsv1.120. As shown in Fig. S4, 148 *BnaLEA*s were unevenly distributed on the An-(73) and Cn-chromosomes (75) with gene number ranging from 2 (Bna_C06) to 17 (Bna_C03). Similarly, the distribution of *LEA* genes on the chromosomes of *B.rapa* and *B. oleracea* were similar as the distribution of orthologous genes *BnaLEA* genes on the An- and Cn-chromosomes in *B. napus* (Fig. S4).

### *Cis*-regulatory elements analysis of the *BnaLEA* promotors

To understand the potential regulatory mechanisms of *BnaLEAs*, *cis*-regulatory elements were analyzed using PlantCARE. A total of 4097 elements were predicted in the promoters of 148 *BnaLEAs* (Fig. S5 and Table S2). There are 1550, 2244, 120 and 183 *cis*-regulatory elements related to plant hormone response, environmental stress response, organ development, and transcription-factor binding sites, respectively (Fig. S5 and Table S2). Among them, the light-responsive, ABA-responsive, and MeJA-responsive *cis*-regulatory elements were the top three. The result indicated that most *BnaLEAs* could be regulated by various plant hormones and environmental stresses in rapeseed.

### Expression profiling of the *BnaLEA* genes in different tissues

**Fig. 2:**
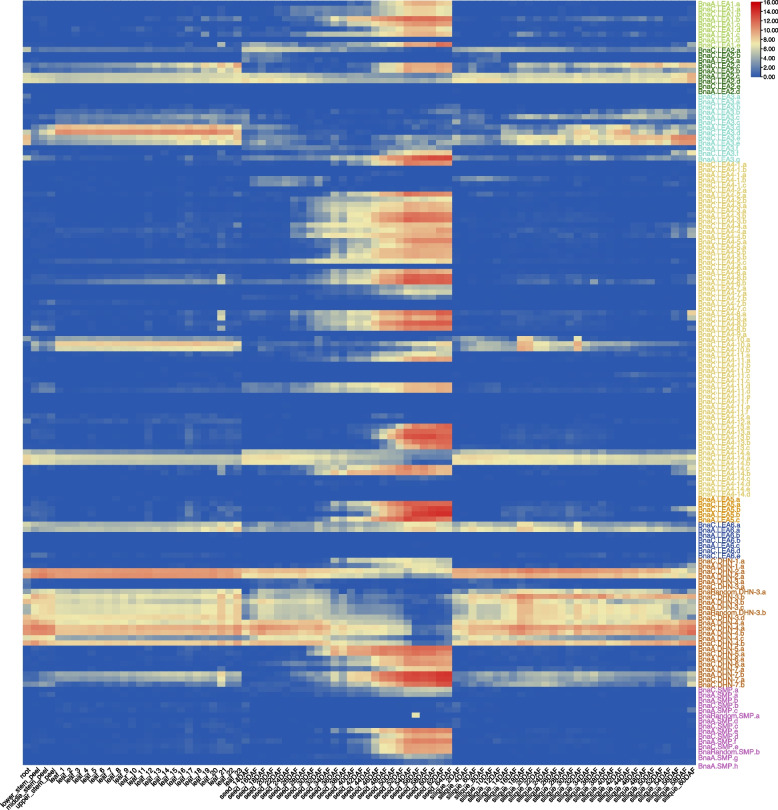
Expression of *BnaLEA* genes in different tissues and organs of ZS11 (semi-winter type cv. Zhongshuang 11). Colored rectangles indicate expression levels of *BnaLEA* genes. Red means high expression, Blue means low expression. Color from red to blue represents descending log_2_(TPM + 1)

To investigate the expression patterns of the *BnaLEA* genes in different tissues, their expression patterns in five different tissues/organs of rapeseed (root, stem, leaf, seed, and silique) were determined by analyzing RNA-Seq data (Table S3) [38]. Most of the *BnaLEA* genes (96/148) were highly expressed in mature seeds whereas they were lowly expressed (TPM< 1) or even not expressed in the other tissues examined, especially in the root tissue (Fig. 2). Additionally, members of LEA_2 (4/9), LEA_3 (3/13) and DHN (15/25) subgroup members, as well as *BnaA.LEA6.a* and *BnaC.LEA6.a* from LEA_6, were highly expressed across different vegetative and reproductive organs during different development stages (Fig. 2). The differences were that the expression of *BnaA.LEA6.a* and *BnaC.LEA6.a* gene continued to increase and were highly expressed during plant seed development, whereas the expression levels of the remaining genes began to decrease after 50 days of seed development, with TPM values trending towards 0, such as *BnaA.LEA2.c* in the LEA_2 subgroup, *BnaC.LEA3.d* in the LEA_3, and *BnaRandom.DHN-3.b* in the DHN subgroup (Fig. 2).

### Expression profiling of the *BnaLEA* genes under different abiotic stresses

**Fig. 3:**
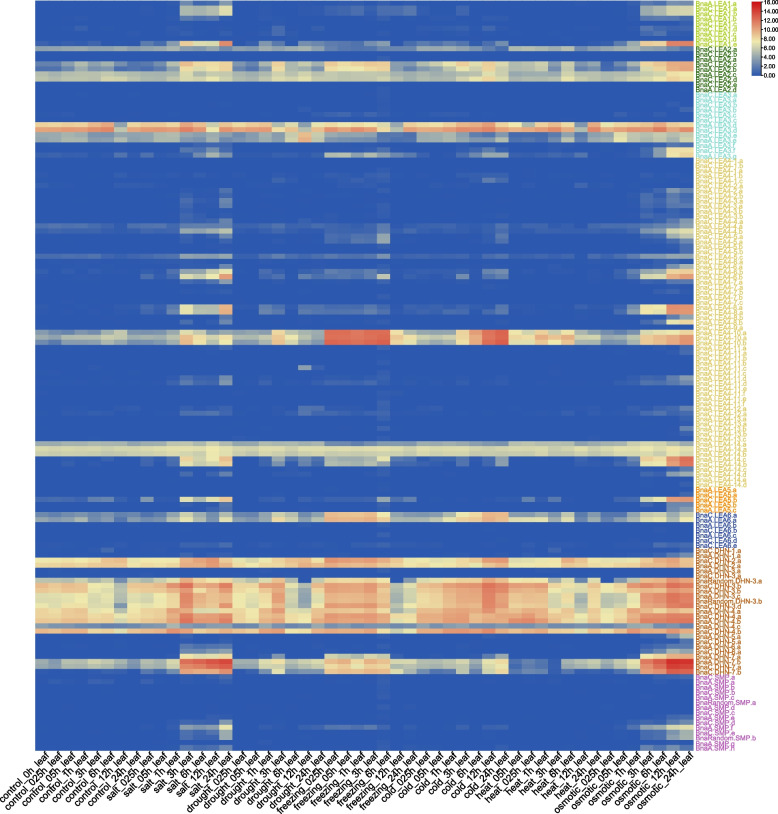
Expression of *BnaLEA* genes under different abiotic stresses treatments in leaves. Colored rectangles indicate expression levels of *BnaLEA* genes. Red means high expression, Blue means low expression. Color from red to blue represents descending log_2_(TPM + 1)

To investigate the expression patterns of the *BnaLEA* genes under different abiotic stresses, their expression patterns under stresses (salt, drought, freezing, cold, heat, and osmotic) were identified by analyzing RNA-Seq data (Table S3). Members of the *BnaLEA* gene family exhibited similar expression patterns under different stresses in roots and leaves (Fig. 3, and Fig. S6). The expression of most *BnaLEA* genes was up regulated by salt stress, freezing stress, and osmotic stress. Additionally, some genes were particularly responsive to freezing stress in both roots and leaves (Fold Change > 10, TPM > 1). Examples included some members from the LEA_4 subgroup (*BnaA.LEA4-10.a*, *BnaA.LEA4-10.b* and *BnaC.LEA4-10.a*), and the LEA_6 subgroup (*BnaA.LEA6.a* and *BnaC.LEA6.a*).

### Expression profiling of the *BnaLEA* genes under different phytohormone treatments

**Fig. 4:**
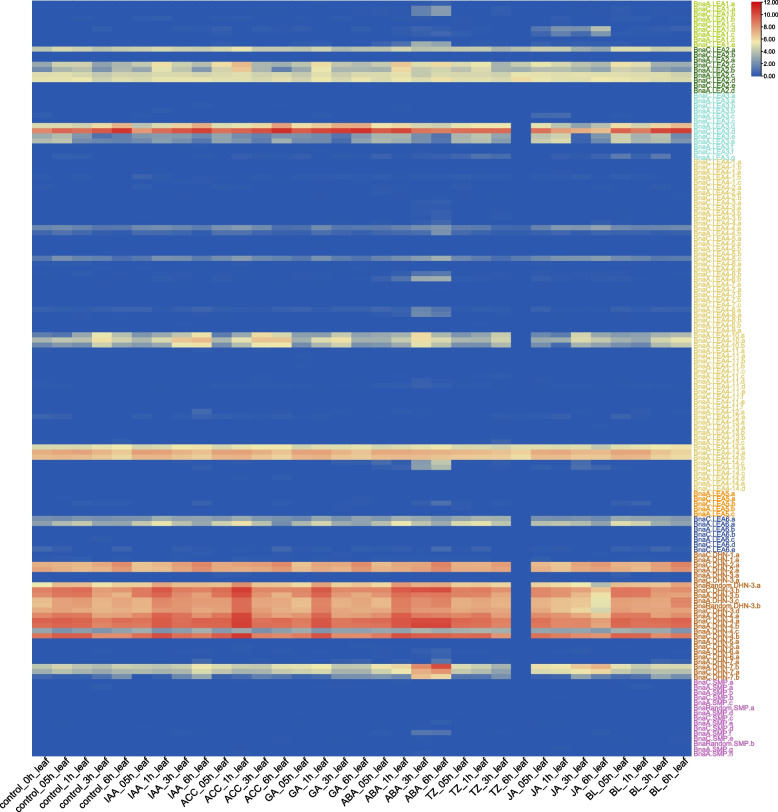
Expression patterns of *BnaLEA* genes under different plant hormones treatments in leaves. Colored rectangles indicate expression levels of *BnaLEA* genes. Red means high expression, Blue means low expression. Color from red to blue represents descending log_2_(TPM + 1)

To investigate the expression patterns of the *BnaLEA* genes under different phytohormone treatments, their expression patterns under phytohormone treatments (indole-3-acetic acid, IAA; 1-aminocyclopropane-1-carboxylic acid, ACC; gibberellic acid, GA; abscisic acid, ABA; cytokinin, TZ; jasmonate, JA; and brassinolide, BL) were determined by analyzing RNA-Seq data (Table S3). A few LEA subgroup members were strongly up-regulated induced by different hormones-treatments (Fold Change > 2 and TPM > 1), and they exhibited distinct expression patterns in leaves and roots (Fig. 4 and Fig. S7). Notably, the *LEA* genes demonstrated a more pronounced response to ABA both in leaves and roots (Fig. 4 and Fig. S7). Less predictably, most LEA subgroup members showed slightly or no response to hormones-treatments in leaves (99/148) and roots (76/148) (Fig. 4 and Fig. S7).

**Fig. 5:**
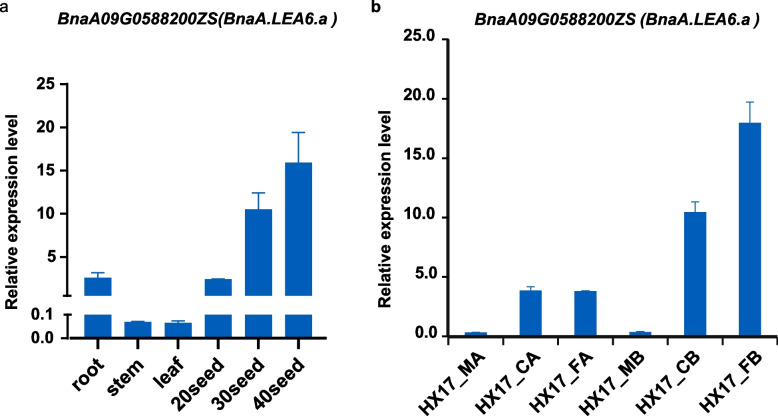
qRT-PCR analysis of *BnaA.LEA6.a* gene in different tissues/organs and under cold and freezing stress. **a** The expression levels of the *BnaA.LEA6.a* gene in the root, stem, leaf, and seeds (at 20, 30, and 40 days after flowering) in the *Brassica napus* variety Zhongshuang 6. **b** The expression levels of *BnaA.LEA6.a* gene under cold (4℃) and freezing (-4℃) stress in *B. napus*. In group A, after 14 days of cold acclimation, plants were subjected to 12 h of low temperature at 4℃ and freezing stress at -4℃. In group B, plants were not cold-acclimated and were directly subjected to 12 h of treatment at 4℃ and freezing stress at -4℃. *BnaActin* (*BnaA09G0588200ZS*) was used as the endogenous reference gene. The relative transcript levels were averaged over the three technical replicates

The LEA genes are typically expressed at high levels in the late stages of plant seed development. In contrast, the results of RNA-Seq analysis revealed that the *BnaA.LEA6.a* gene exhibited high expression levels in various plant tissues at different developmental stages and showed a strong response to freezing stress. To validate the results of transcriptome data, we performed qRT-PCR to detect the transcript levels of the *BnaA.LEA6.a* gene in different tissues (root, stem, leaf, and seed) of rapeseed and under freezing stress treatments (Fig. 5a, b). The *BnaA.LEA6.a* gene was expressed in the roots, stems, and leaves of the plant, especially exhibiting high expression levels during 40-day-old seeds (Fig. 5a). Additionally, the expression of the *BnaA.LEA6.a* gene was significantly up-regulated under freezing stress, with or without cold acclimation (Fig. 5b).

### Overexpression of *BnaA.LEA6.a* enhances freezing tolerance in rapeseed

**Fig. 6:**
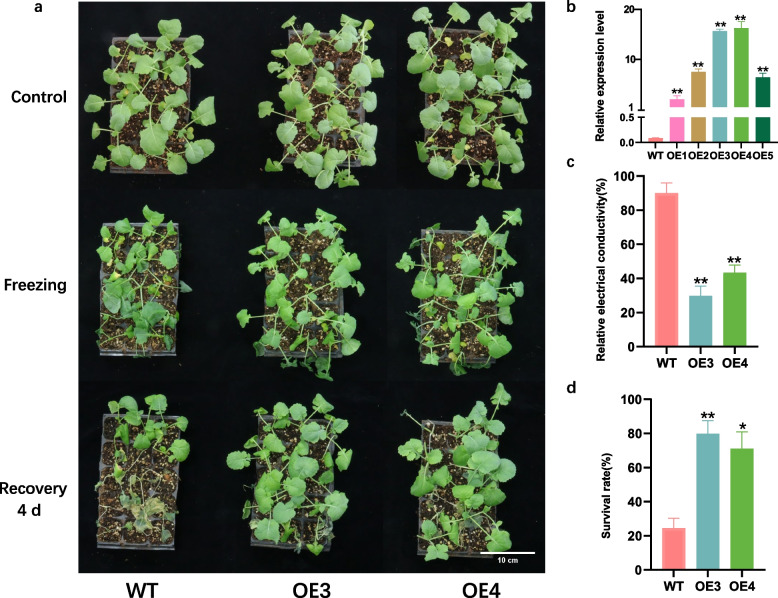
Phenotypes of the *BnaA.LEA6.a*-overexpression transgenic rapeseed lines and WT before and after freezing stress treatment. **a** 30-day-old WT and OE lines recovered to 4 d phenotype after 2 h treatment at -3.5 ℃. **b** The relative expression level of *BnaA.LEA6.a*-overexpression transgenic plants and WT. **c** The relative electrical conductivity (%) after freezing treatment. **d** Statistics on the survival rate of 30-day-old WT and OE lines treated at -3.5 ℃ for 2 h and recovered for 7 d. Data were expressed as the mean of triplicate values, and the error bar represented the SEM. Asterisks indicate statistically significant differences between transgenic lines and the corresponding WT plants at *P* ≤ 0.01(**), *P* ≤ 0.05(*)

To further investigate the function of the *BnaA.LEA6.a* gene under freezing stress, we constructed a 35S: *BnaA.LEA6.a* vector and obtained six *BnaA.LEA6.a* overexpression lines in Zhongshuang 6. Among them, two lines (OE3 and OE4) with the highest expression were selected for freezing treatment (Fig. 6a, b). After freezing treatment at -3.5 ℃ for 2 h, the *BnaA.LEA6.a* overexpression lines showed only partial leaf icing and water-soaking, while most of the WT exhibited severe icing and water-soaking throughout the whole plant, accompanied by plant softening (Fig. 6a). Additionally, the relative electrical conductivity of the overexpression lines was significantly lower than that of the WT (Fig. 6c). Following seven days of recovery, the *BnaA.LEA6.a* overexpression plants showed significantly better growth and higher survival rates compared to the WT plants (Fig. 6a, d). These results indicate that *BnaA.LEA6.a* can enhance the freezing tolerance of rapeseed.

## Discussion

Low temperatures have detrimental effects on plant growth and even result in plant death. It is imperative to explore genes that enhance the low-temperature tolerance in rapeseed. Numerous studies have shown the positive impact of *LEA* gene expression on stress tolerance in several crops, such as rice [39], maize [40], wheat [41], and rapeseed [42]. Nevertheless, there is a scarcity of reports specifically focusing on the role of *LEA* genes in conferring freezing tolerance in rapeseed.

In this study, 306 *LEA* genes were identified in *B. rapa*, *B. oleracea* and *B. napus* and can be divided into eight subgroups (Fig. 1). The LEA_4 subgroup having the highest number of members, consistent with previous studies in *Arabidopsis* [43] and *B. napus* [44]. Despite the limited sequence similarity among members within the LEA_4 subgroup, as indicated by their distribution across multiple branches, they all share the characteristic Pfam structural domain (Table S1 and Fig. 1). In general, LEA proteins are relatively small, but some larger LEA proteins (~ 80.92805 kDa) were found in the LEA_4 subgroup, which was consistent with the research on LEA proteins in *Arabidopsis* [43]. Previous studies in *Arabidopsis* and *Oryza sativa* have shown that multi-stimuli response genes were shorter and had fewer introns [45, 46]. In this study, 97.97% (145/148) of *BnaLEA* genes contained only 0–2 introns, of which only 10 had a DNA length longer than 1800 bp (Table S1 and Fig. S2). Under different adversity treatment in leaves, 62.07% (90/145) of the gene family members exhibited varying levels of adversity-induced expression (TPM > 1), with only 1 gene having a length greater than 1800 bp and fewer than 2 introns. Curiously, the CDS length and the number of exons and introns of the LEA_4 subgroup members *BnaC.LEA4-11.b* and *BnaC.LEA4-11.e* genes were similar to their homologous genes *BolC.LEA4-11.a* and *BolC.LEA4-11.d* genes, but these two genes had very long introns. We analyzed the gene annotation of the *BnaC.LEA4-11.b* and *BnaC.LEA4-11.e* genes in different rapeseed varieties. ( Zhongshuang11, Westar and Damor). The analysis results showed that these genes exhibited similar genomic structures with a significant presence of transposons in the Zhongshuang11 and Damor varieties. However, the relevant annotations for these genes were not found in the Westar variety, which is possibly due to the presence of a large number of transposons leading to gene instability and variation between different varieties. Correspondingly, the promoter sequences of LEA_6 members were rich in light-responsive and defense hormone-responsive *cis*-regulatory elements, and LEA_6 members were induced by abiotic stress and hormones-treatments (Table S2 and Fig. S5).

It can obtain clues from gene expression patterns to explore function of genes [47]. Typical *LEA* genes exhibited at high expression levels in the late stages of seed development in *Arabidopsis* [43] and *Oryza sativa* [39], Similarly, we found that most of the *BnaLEA* gene family members are also highly expressed in the late stage of seed development (Table S3 and Fig. 2). However, there also existed a portion of genes, such as *BnaC.LEA2.c* and *BnaA.LEA2. b* from LEA_2 subgroup, and 10 members from DHN subgroup, that were instead highly expressed in the early stages of seed development and other tissues (Table S3 and Fig. 2), which suggested the diverse functions of those *LEAs*. It was found that most *BnaLEA* genes were induced to be up-regulated by salt stress, freezing stress, and osmotic stress. however, the expression levels of these genes were relatively low in plant tissues. In contrast, the *BnaA.LEA6.a* gene showed strong responses to freezing stress in both roots and leaves, with its expression levels continuing to increase across various developmental stages of plant seeds. These results implied that the *BnaA.LEA6.a* gene may play an important role in the growth, development, and response to freezing stress in rapeseed. The qRT-PCR results further confirmed that the expression levels of the *BnaA.LEA6.a* gene remained high throughout seed development and increased under freezing stress conditions.

Genes from the same family may have different roles in regulating growth and development under abiotic or biotic stress. Previous studies had shown that overexpression of the *BnLEA57* (named *BnaC.LEA4-8.a*/*BnaC05G0444500ZS* in this study) gene or its homolog *BnLEA55* (named *BnaA.LEA4-8.a* /*BnaA05G0396800ZS* in this study) in *Arabidopsis* increased seed oil content [48]. In rice, the overexpression of *OsEm1*, which encoded a group I LEA protein, had been shown to increase the survival rate of rice plants under drought stress during nutritional stages [49]. *MsLEA* (LEA_4, *AT4G13230*) recruited and protected its target proteins (SOD and Ms1770) and increased alfalfa tolerance against drought and aluminum stresses [50]. The *OsLEA9* gene in rice negatively regulates cold tolerance during the reproductive and seedling stages [51]. Here, we identified the *BnaA.LEA6.a* gene from the LEA_6 subgroup, which strongly responds to freezing stress, and verified its function by assessing the freezing tolerance of transgenic rapeseed at the seedling stage. However, the molecular mechanism by which *BnaA.LEA6.a* confers freezing tolerance in rapeseed needs to be further analyzed.

## Conclusion

In this study, 306 *LEA* genes were identified in *B. rapa, B. oleracea and B. napus*, which were categorized into eight subgroups. 71.57% (219/306) of *BnaLEA* genes contained 0–1 introns. Most *cis*-regulatory elements in the promotors of *BnaLEA* were related to plant hormone and environmental stress response. *BnaLEA* genes displayed different spatiotemporal expression patterns, as well as various abiotic stress and hormone responsive expression patterns. Most of them were up-regulated induced by salt stress, freezing stress, and osmotic stress. Overexpression of *BnaA.LEA6.a* resulted in an increased freezing tolerance in rapeseed. This study contributes to our understanding of LEA genes in rapeseed and offers an ideal candidate gene (*BnaA.LEA6.a*) for molecular breeding of freezing-tolerant rapeseed cultivars.

### Supplementary Information

Supplementary Material 1: Supplementary Table 1. Characteristics of *LEAs* in *B.* *rapa*, *B.* *oleracea*, and *B.* *napus*.

Supplementary Material 2: Supplementary Table 2. *Cis*-regulatory elements in promotors of *BnaLEAs*.

Supplementary Material 3: Supplementary Table 3. RNA-seq data of expression levels of *BnaLEAs* in the different tissues and under different abiotic stresses and plant hormone treatments in leaves and roots.

Supplementary Material 4: Supplementary Table 4. List of primers used in this study.

Supplementary Material 5: Supplementary Figure 1. MEME analysis of LEA proteins. Four motifs in *B. rapa*, *B. oleracea*, *B. napus* and *Arabidopsis*. LEA proteins were identified by MEME and were represented by four boxes of different colors.

Supplementary Material 6: Supplementary Figure 2. Gene structures of *LEA* genes analyzed by TBtools. Green boxes, black lines, and orange boxes indicate untranslated regions, introns, CDS, respectively.

Supplementary Material 7: Supplementary Figure 3. Syntenic relationships among *LEA* genes of *B.* *napus*, *B.* *rapa*, *B.* *oleracea* and *Arabidopsis*. The chromosomes of *B.* *napus*, *B.* *rapa*, *B.* *oleracea* and *Arabidopsis* were shown in green, purple, and pink, and blue respectively. The Bna_Random, A0_Random and C0_Random chromosome fragment is very short and is not marked in the figure. The orthologous and paralogous *LEA* genes were mapped onto the chromosomes/scaffolds and linked with each other. The syntenic* LEA *gene pairs from *B.* *rapa* and *Arabidopsis*, *B.* *oleracea* and *Arabidopsis*, *B. napus* and *Arabidopsis*, *B. rapa* and *B.* *oleracea*, *B.* *rapa* and *B. napus*, *B.* *oleracea* and *B.* *napus*, were linked by pink, orange, green, gold, red, and blue lines, respectively.

Supplementary Material 8: Supplementary Figure 4. Distribution of *LEA *genes on the *B.* *rapa* (A), *B.* *oleracea* (B) and *B.* *napus* (C) chromosomes. (A) A01–10: *B.* *rapa* chromosomes, Scaffold000300, Scaffold000111 and Scaffold000435: unanchored scaffolds from *B.* *rapa*; (B) C1–9: *B.* *oleracea* chromosomes, Scaffold24830, Scaffold01143 and Scaffold13604: unanchored scaffolds from *B.* *oleracea*; (C) Bna_A01–10: *B.* *napus* An-subgenome chromosomes; Bna_C01–09: *B.* *napus* Cn-subgenome chromosomes; scaffold0510, scaffold0026 and scaffold0327: unanchored scaffolds from *B. napus.*

Supplementary Material 9: Supplementary Figure 5. The *cis*-regulatory elements in the promoters of *BnaLEAs* predicted by PlantCARE. Boxes filled with different colors represent different *cis*-regulatory elements.

Supplementary Material 10: Supplementary Figure 6. Expression of *BnaLEA* genes under different abiotic stresses in roots. Colored rectangles indicate expression levels of *BnaLEA* genes. Red means high expression, Blue means low expression. Color from red to blue represents descending log_2_(TPM+1).

Supplementary Material 11: Supplementary Figure 7. Expression of *BnaLEA* genes under hormones treatments in roots. Colored rectangles indicate expression levels of *BnaLEA* genes. Red means high expression, Blue means low expression. Color from red to blue represents descending log_2_(TPM+1).

## Data Availability

Data is provided within the manuscript or supplementary information files.
