# Supplementary material for: Genome-wide characterization of LEA gene family reveals a positive role of BnaA.LEA6.a in freezing tolerance in rapeseed (Brassica napus L.)
Source: BMC Plant Biol. 2024 May 21;24:433. doi: 10.1186/s12870-024-05111-7 (PMC11106994; doi:10.1186/s12870-024-05111-7)
Supplement: Supplementary file 5 — Supplementary Material 5: Supplementary Figure 1. MEME analysis of LEA proteins. Four motifs in B. rapa, B. oleracea, B. napus and Arabidopsis. LEA proteins were identified by MEME and were represented by four boxes of different colors. [file 12870_2024_5111_MOESM5_ESM.pdf]

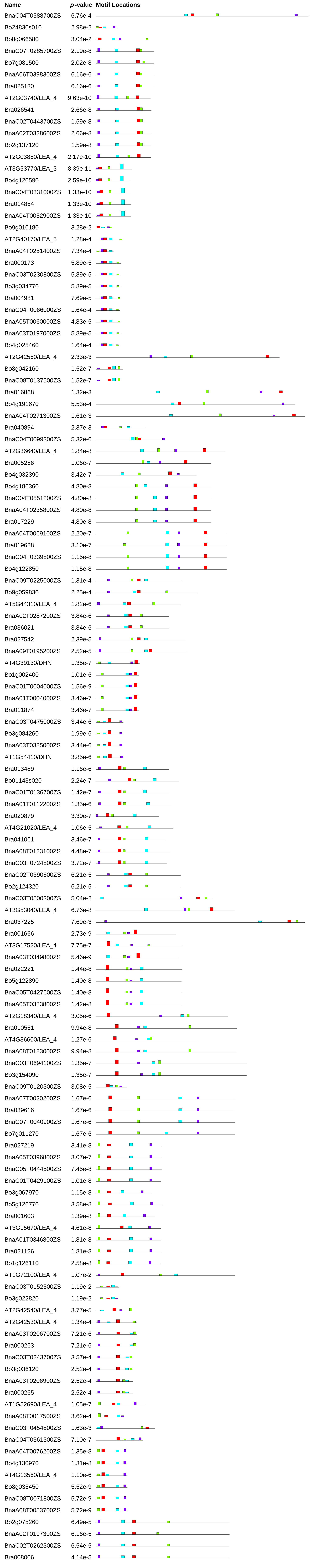
Name p-value Motif Locations
BnaC04T0588700ZS 6.76e-4
Bo24830s010 2.98e-2
Bo8g066580 3.04e-2
BnaC07T0285700ZS 2.19e-8
Bo7g081500 2.02e-8
BnaA06T0398300ZS 6.16e-6
Bra025130 6.16e-6
AT2G03740/LEA_4 9.63e-10
Bra026541 2.66e-8
BnaC02T0443700ZS 1.59e-8
BnaA02T0328600ZS 2.66e-8
Bo2g137120 1.59e-8
AT2G03850/LEA_4 2.17e-10
AT3G53770/LEA_3 8.39e-11
Bo4g120590 2.59e-10
BnaC04T0331000ZS 1.33e-10
Bra014864 1.33e-10
BnaA04T0052900ZS 1.33e-10
Bo9g010180 3.28e-2
AT2G40170/LEA_5 1.28e-4
BnaA04T0251400ZS 7.34e-4
Bra000173 5.89e-5
BnaC03T0230800ZS 5.89e-5
Bo3g034770 5.89e-5
Bra004981 7.69e-5
BnaC04T0066000ZS 1.64e-4
BnaA05T0060000ZS 4.83e-5
BnaA03T0197000ZS 5.89e-5
Bo4g025460 1.64e-4
AT2G42560/LEA_4 2.33e-3
Bo8g042160 1.52e-7
BnaC08T0137500ZS 1.52e-7
Bra016868 1.32e-3
Bo4g191670 5.53e-4
BnaA04T0271300ZS 1.61e-3
Bra040894 2.37e-3
BnaC04T0099300ZS 5.32e-6
AT2G36640/LEA_4 1.84e-8
Bra005256 1.06e-7
Bo4g032390 3.42e-7
Bo4g186360 4.80e-8
BnaC04T0551200ZS 4.80e-8
BnaA04T0235800ZS 4.80e-8
Bra017229 4.80e-8
BnaA04T0069100ZS 2.20e-7
Bra019628 3.10e-7
BnaC04T0339800ZS 1.15e-8
Bo4g122850 1.15e-8
BnaC09T0225000ZS 1.31e-4
Bo9g059830 2.25e-4
AT5G44310/LEA_4 1.82e-6
BnaA02T0287200ZS 3.84e-6
Bra036021 3.84e-6
Bra027542 2.39e-5
BnaA09T0195200ZS 2.52e-5
AT4G39130/DHN 1.35e-7
Bo1g002400 1.01e-6
BnaC01T0004000ZS 1.56e-9
BnaA01T0004000ZS 3.46e-7
Bra011874 3.46e-7
BnaC03T0475000ZS 3.44e-6
Bo3g084260 1.99e-6
BnaA03T0385000ZS 3.44e-6
AT1G54410/DHN 3.85e-6
Bra013489 1.16e-6
Bo01143s020 2.24e-7
BnaC01T0136700ZS 1.42e-7
BnaA01T0112200ZS 1.35e-6
Bra020879 3.30e-7
AT4G21020/LEA_4 1.06e-5
Bra041061 3.46e-7
BnaA08T0123100ZS 4.48e-7
BnaC03T0724800ZS 3.72e-7
BnaC02T0390600ZS 6.21e-5
Bo2g124320 6.21e-5
BnaC03T0500300ZS 5.04e-2
AT3G53040/LEA_4 6.76e-8
Bra037225 7.69e-3
Bra001666 2.73e-9
AT3G17520/LEA_4 7.75e-7
BnaA03T0349800ZS 5.46e-9
Bra022221 1.44e-8
Bo5g122890 1.40e-8
BnaC05T0427600ZS 1.40e-8
BnaA05T0383800ZS 1.42e-8
AT2G18340/LEA_4 3.05e-6
Bra010561 9.94e-8
AT4G36600/LEA_4 1.27e-6
BnaA08T0183000ZS 9.94e-8
BnaC03T0694100ZS 1.35e-7
Bo3g154090 1.35e-7
BnaC09T0120300ZS 3.08e-5
BnaA07T0020200ZS 1.67e-6
Bra039616 1.67e-6
BnaC07T0040900ZS 1.67e-6
Bo7g011270 1.67e-6
Bra027219 3.41e-8
BnaA05T0396800ZS 3.07e-7
BnaC05T0444500ZS 7.45e-8
BnaC01T0429100ZS 1.01e-8
Bo3g067970 1.15e-8
Bo5g126770 3.58e-8
Bra001603 1.39e-8
AT3G15670/LEA_4 4.61e-8
BnaA01T0346800ZS 1.81e-8
Bra021126 1.81e-8
Bo1g126110 2.58e-8
AT1G72100/LEA_4 1.07e-2
BnaC03T0152500ZS 1.19e-2
Bo3g022820 1.19e-2
AT2G42540/LEA_4 3.77e-5
AT2G42530/LEA_4 1.34e-4
BnaA03T0206700ZS 7.21e-6
Bra000263 7.21e-6
BnaC03T0243700ZS 3.57e-4
Bo3g036120 2.52e-4
BnaA03T0206900ZS 2.52e-4
Bra000265 2.52e-4
AT1G52690/LEA_4 1.05e-7
BnaA08T0017500ZS 3.62e-4
BnaC03T0454800ZS 1.63e-3
BnaC04T0361300ZS 7.10e-7
BnaA04T0076200ZS 1.35e-8
Bo4g130970 1.31e-8
AT4G13560/LEA_4 1.10e-6
Bo8g035450 5.52e-9
BnaC08T0071800ZS 5.72e-9
BnaA08T0053700ZS 5.72e-9
Bo2g075260 6.49e-5
BnaA02T0197300ZS 6.16e-5
BnaC02T0262300ZS 6.54e-5
Bra008006 4.14e-5

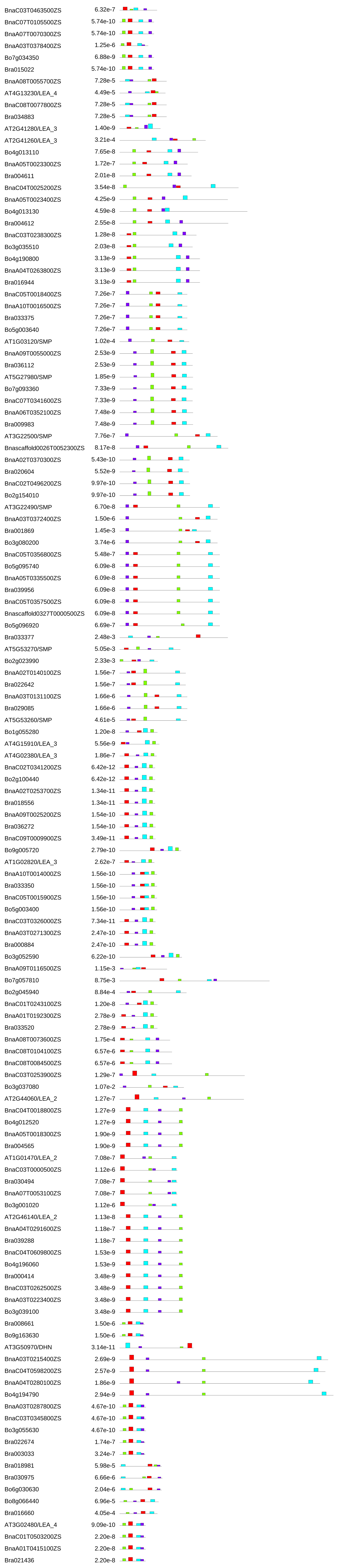
BnaC03T0463500ZS 6.32e-7
BnaC07T0105500ZS 5.74e-10
BnaA07T0070300ZS 5.74e-10
BnaA03T0378400ZS 1.25e-6
Bo7g034350 6.88e-9
Bra015022 5.74e-10
BnaA08T0055700ZS 7.28e-5
AT4G13230/LEA_4 4.49e-5
BnaC08T0077800ZS 7.28e-5
Bra034883 7.28e-5
AT2G41280/LEA_3 1.40e-9
AT2G41260/LEA_3 3.21e-4
Bo4g013110 7.65e-8
BnaA05T0023300ZS 1.72e-7
Bra004611 2.01e-8
BnaC04T0025200ZS 3.54e-8
BnaA05T0023400ZS 4.25e-9
Bo4g013130 4.59e-8
Bra004612 2.55e-8
BnaC03T0238300ZS 1.28e-8
Bo3g035510 2.03e-8
Bo4g190800 3.13e-9
BnaA04T0263800ZS 3.13e-9
Bra016944 3.13e-9
BnaC05T0018400ZS 7.26e-7
BnaA10T0016500ZS 7.26e-7
Bra033375 7.26e-7
Bo5g003640 7.26e-7
AT1G03120/SMP 1.02e-4
BnaA09T0055000ZS 2.53e-9
Bra036112 2.53e-9
AT5G27980/SMP 1.85e-9
Bo7g093360 7.33e-9
BnaC07T0341600ZS 7.33e-9
BnaA06T0352100ZS 7.48e-9
Bra009983 7.48e-9
AT3G22500/SMP 7.76e-7
Bnascaffold0026T0052300ZS 8.17e-8
BnaA02T0370300ZS 5.43e-10
Bra020604 5.52e-9
BnaC02T0496200ZS 9.97e-10
Bo2g154010 9.97e-10
AT3G22490/SMP 6.70e-8
BnaA03T0372400ZS 1.50e-6
Bra001869 1.45e-3
Bo3g080200 3.74e-6
BnaC05T0356800ZS 5.48e-7
Bo5g095740 6.09e-8
BnaA05T0335500ZS 6.09e-8
Bra039956 6.09e-8
BnaC05T0357500ZS 6.09e-8
Bnascaffold0327T0000500ZS 6.09e-8
Bo5g096920 6.69e-7
Bra033377 2.48e-3
AT5G53270/SMP 5.05e-3
Bo2g023990 2.33e-3
BnaA02T0140100ZS 1.56e-7
Bra022642 1.56e-7
BnaA03T0131100ZS 1.66e-6
Bra029085 1.66e-6
AT5G53260/SMP 4.61e-5
Bo1g055280 1.20e-8
AT4G15910/LEA_3 5.56e-9
AT4G02380/LEA_3 1.86e-7
BnaC02T0341200ZS 6.42e-12
Bo2g100440 6.42e-12
BnaA02T0253700ZS 1.34e-11
Bra018556 1.34e-11
BnaA09T0025200ZS 1.54e-10
Bra036272 1.54e-10
BnaC09T0009900ZS 3.49e-11
Bo9g005720 2.79e-10
AT1G02820/LEA_3 2.62e-7
BnaA10T0014000ZS 1.56e-10
Bra033350 1.56e-10
BnaC05T0015900ZS 1.56e-10
Bo5g003400 1.56e-10
BnaC03T0326000ZS 7.34e-11
BnaA03T0271300ZS 2.47e-10
Bra000884 2.47e-10
Bo3g052590 6.22e-10
BnaA09T0116500ZS 1.15e-3
Bo7g057810 8.75e-3
Bo2g045940 8.84e-4
BnaC01T0243100ZS 1.20e-8
BnaA01T0192300ZS 2.78e-9
Bra033520 2.78e-9
BnaA08T0073600ZS 1.75e-4
BnaC08T0104100ZS 6.57e-6
BnaC08T0084500ZS 6.57e-6
BnaC03T0253900ZS 1.29e-7
Bo3g037080 1.07e-2
AT2G44060/LEA_2 1.27e-7
BnaC04T0018800ZS 1.27e-9
Bo4g012520 1.27e-9
BnaA05T0018300ZS 1.90e-9
Bra004565 1.90e-9
AT1G01470/LEA_2 7.08e-7
BnaC03T0000500ZS 1.12e-6
Bra030494 7.08e-7
BnaA07T0053100ZS 7.08e-7
Bo3g001020 1.12e-6
AT2G46140/LEA_2 1.13e-8
BnaA04T0291600ZS 1.18e-7
Bra039288 1.18e-7
BnaC04T0609800ZS 1.53e-9
Bo4g196060 1.53e-9
Bra000414 3.48e-9
BnaC03T0262500ZS 3.48e-9
BnaA03T0223400ZS 3.48e-9
Bo3g039100 3.48e-9
Bra008661 1.50e-6
Bo9g163630 1.50e-6
AT3G50970/DHN 3.14e-11
BnaA03T0215400ZS 2.69e-9
BnaC04T0598200ZS 2.57e-9
BnaA04T0280100ZS 1.86e-9
Bo4g194790 2.94e-9
BnaA03T0287800ZS 4.67e-10
BnaC03T0345800ZS 4.67e-10
Bo3g055630 4.67e-10
Bra022674 1.74e-7
Bra003033 3.24e-7
Bra018981 5.98e-5
Bra030975 6.66e-6
Bo6g030630 2.04e-6
Bo8g066440 6.96e-5
Bra016660 4.05e-4
AT3G02480/LEA_4 9.09e-10
BnaC01T0503200ZS 2.20e-8
BnaA01T0415100ZS 2.20e-8
Bra021436 2.20e-8

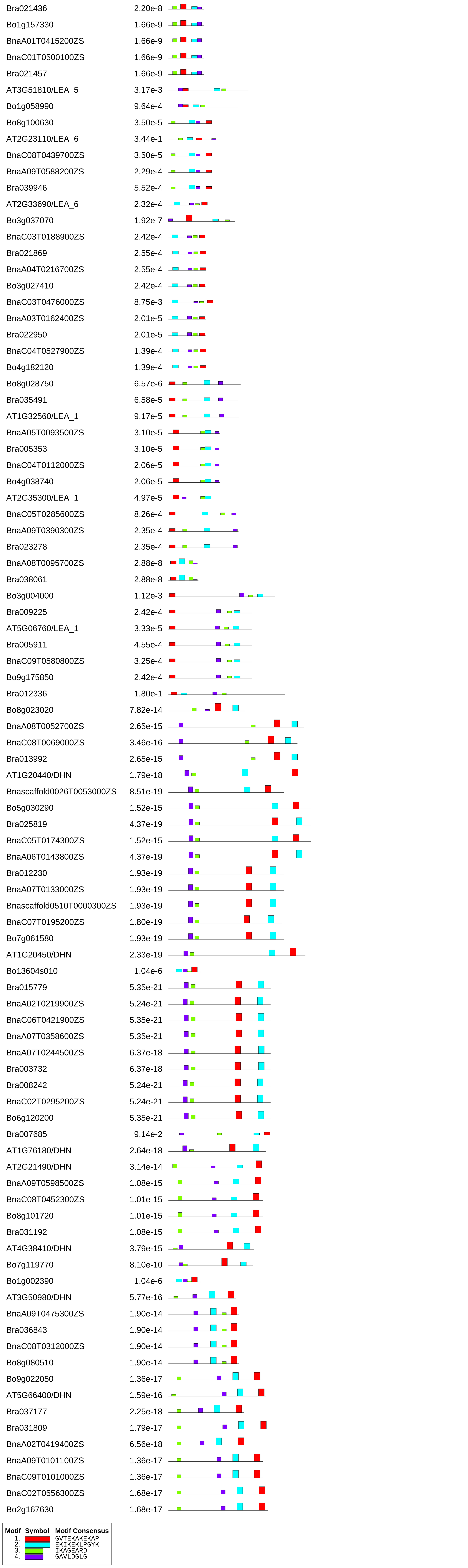
Bra021436 2.20e-8
Bo1g157330 1.66e-9
BnaA01T0415200ZS 1.66e-9
BnaC01T0500100ZS 1.66e-9
Bra021457 1.66e-9
AT3G51810/LEA_5 3.17e-3
Bo1g058990 9.64e-4
Bo8g100630 3.50e-5
AT2G23110/LEA_6 3.44e-1
BnaC08T0439700ZS 3.50e-5
BnaA09T0588200ZS 2.29e-4
Bra039946 5.52e-4
AT2G33690/LEA_6 2.32e-4
Bo3g037070 1.92e-7
BnaC03T0188900ZS 2.42e-4
Bra021869 2.55e-4
BnaA04T0216700ZS 2.55e-4
Bo3g027410 2.42e-4
BnaC03T0476000ZS 8.75e-3
BnaA03T0162400ZS 2.01e-5
Bra022950 2.01e-5
BnaC04T0527900ZS 1.39e-4
Bo4g182120 1.39e-4
Bo8g028750 6.57e-6
Bra035491 6.58e-5
AT1G32560/LEA_1 9.17e-5
BnaA05T0093500ZS 3.10e-5
Bra005353 3.10e-5
BnaC04T0112000ZS 2.06e-5
Bo4g038740 2.06e-5
AT2G35300/LEA_1 4.97e-5
BnaC05T0285600ZS 8.26e-4
BnaA09T0390300ZS 2.35e-4
Bra023278 2.35e-4
BnaA08T0095700ZS 2.88e-8
Bra038061 2.88e-8
Bo3g004000 1.12e-3
Bra009225 2.42e-4
AT5G06760/LEA_1 3.33e-5
Bra005911 4.55e-4
BnaC09T0580800ZS 3.25e-4
Bo9g175850 2.42e-4
Bra012336 1.80e-1
Bo8g023020 7.82e-14
BnaA08T0052700ZS 2.65e-15
BnaC08T0069000ZS 3.46e-16
Bra013992 2.65e-15
AT1G20440/DHN 1.79e-18
Bnascaffold0026T0053000ZS 8.51e-19
Bo5g030290 1.52e-15
Bra025819 4.37e-19
BnaC05T0174300ZS 1.52e-15
BnaA06T0143800ZS 4.37e-19
Bra012230 1.93e-19
BnaA07T0133000ZS 1.93e-19
Bnascaffold0510T0000300ZS 1.93e-19
BnaC07T0195200ZS 1.80e-19
Bo7g061580 1.93e-19
AT1G20450/DHN 2.33e-19
Bo13604s010 1.04e-6
Bra015779 5.35e-21
BnaA02T0219900ZS 5.24e-21
BnaC06T0421900ZS 5.35e-21
BnaA07T0358600ZS 5.35e-21
BnaA07T0244500ZS 6.37e-18
Bra003732 6.37e-18
Bra008242 5.24e-21
BnaC02T0295200ZS 5.24e-21
Bo6g120200 5.35e-21
Bra007685 9.14e-2
AT1G76180/DHN 2.64e-18
AT2G21490/DHN 3.14e-14
BnaA09T0598500ZS 1.08e-15
BnaC08T0452300ZS 1.01e-15
Bo8g101720 1.01e-15
Bra031192 1.08e-15
AT4G38410/DHN 3.79e-15
Bo7g119770 8.10e-10
Bo1g002390 1.04e-6
AT3G50980/DHN 5.77e-16
BnaA09T0475300ZS 1.90e-14
Bra036843 1.90e-14
BnaC08T0312000ZS 1.90e-14
Bo8g080510 1.90e-14
Bo9g022050 1.36e-17
AT5G66400/DHN 1.59e-16
Bra037177 2.25e-18
Bra031809 1.79e-17
BnaA02T0419400ZS 6.56e-18
BnaA09T0101100ZS 1.36e-17
BnaC09T0101000ZS 1.36e-17
BnaC02T0556300ZS 1.68e-17
Bo2g167630 1.68e-17
Motif Symbol Motif Consensus
1. GVTEKAKEKAP
2. EKIKEKLPGYK
3. IKAGEARD
4. GAVLDGLG
