# Supplementary figures and images for: Genome-wide characterization of LEA gene family reveals a positive role of BnaA.LEA6.a in freezing tolerance in rapeseed (Brassica napus L.)
Source: BMC Plant Biol. 2024 May 21;24:433. doi: 10.1186/s12870-024-05111-7 (PMC11106994; doi:10.1186/s12870-024-05111-7)

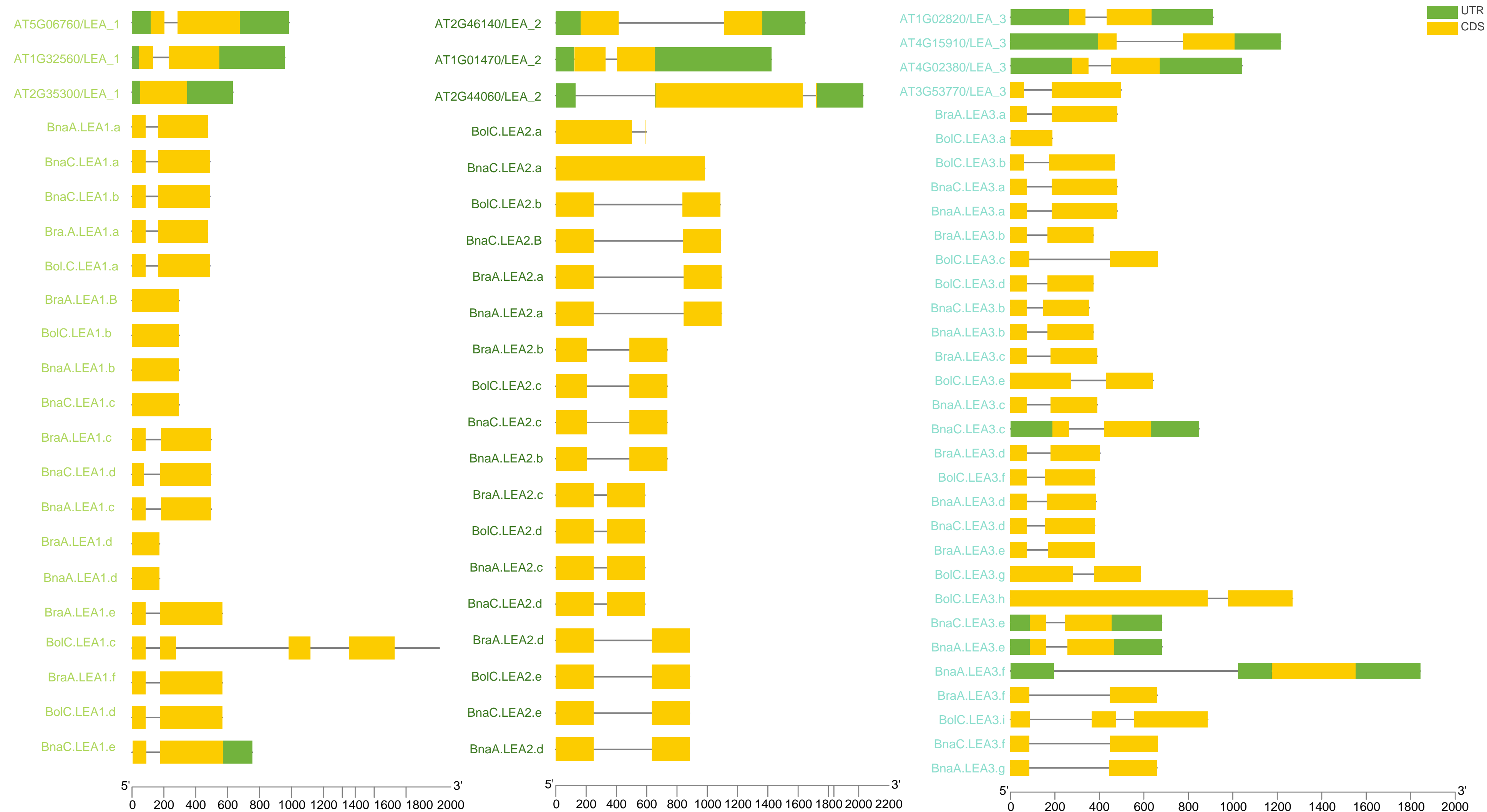

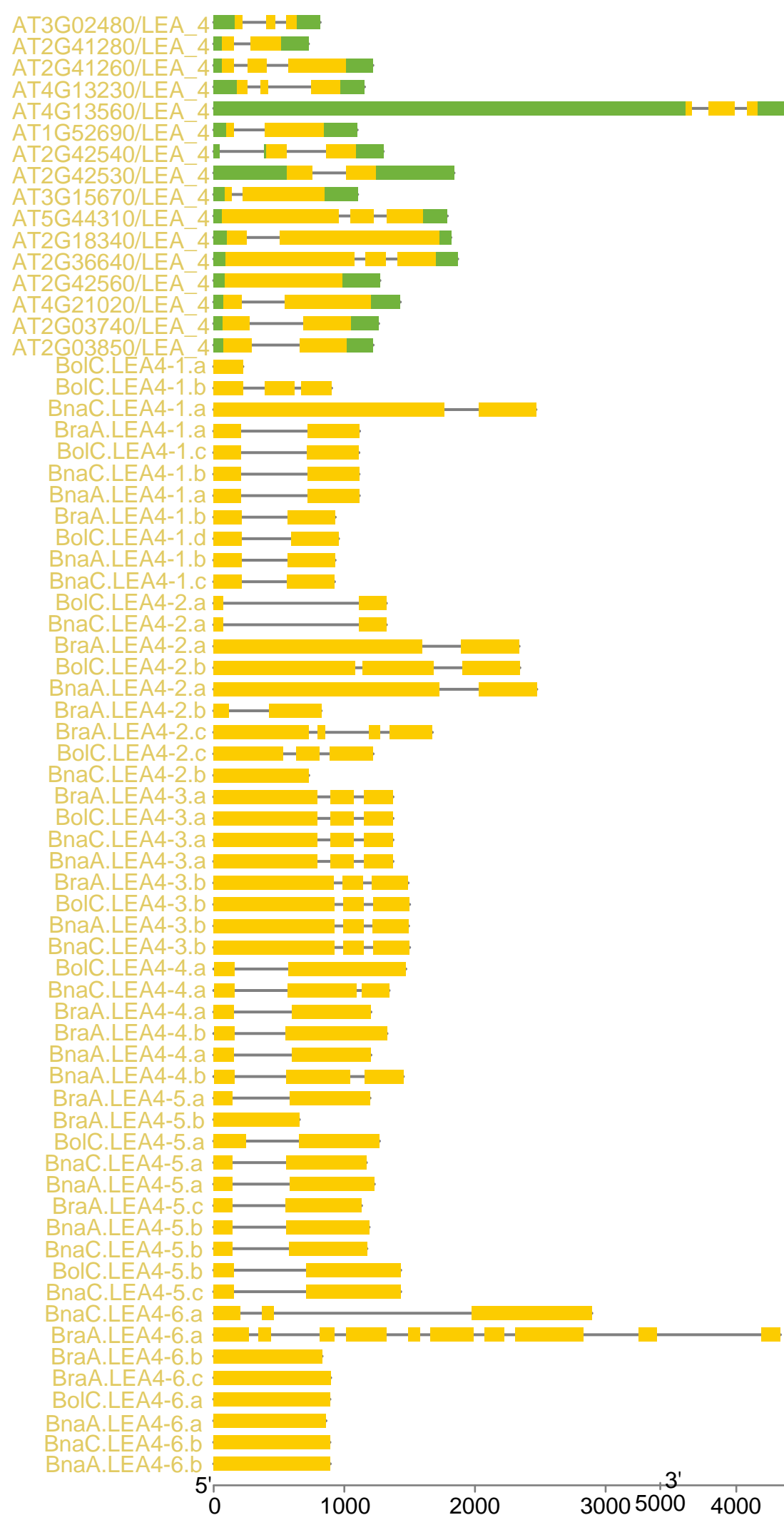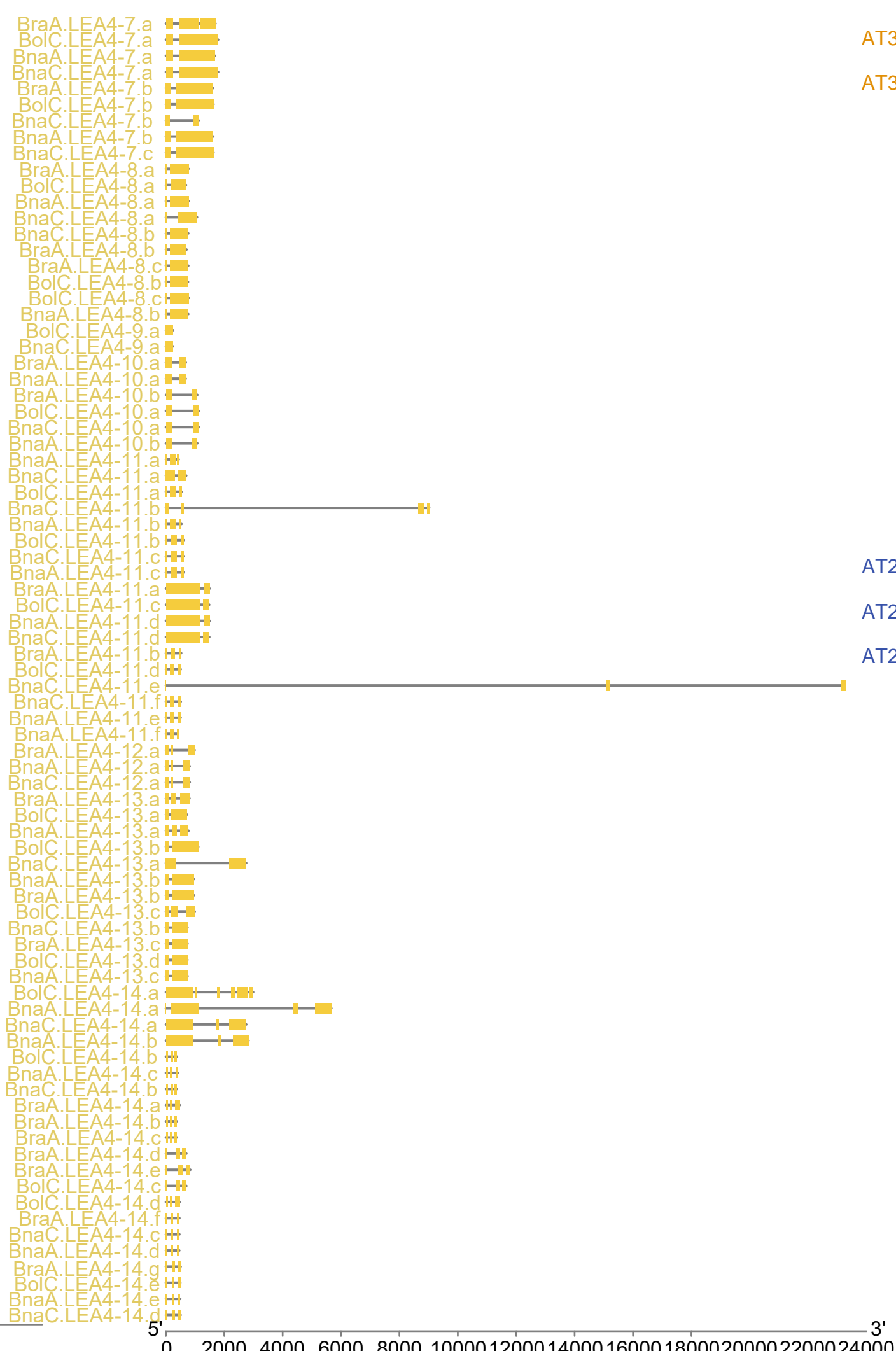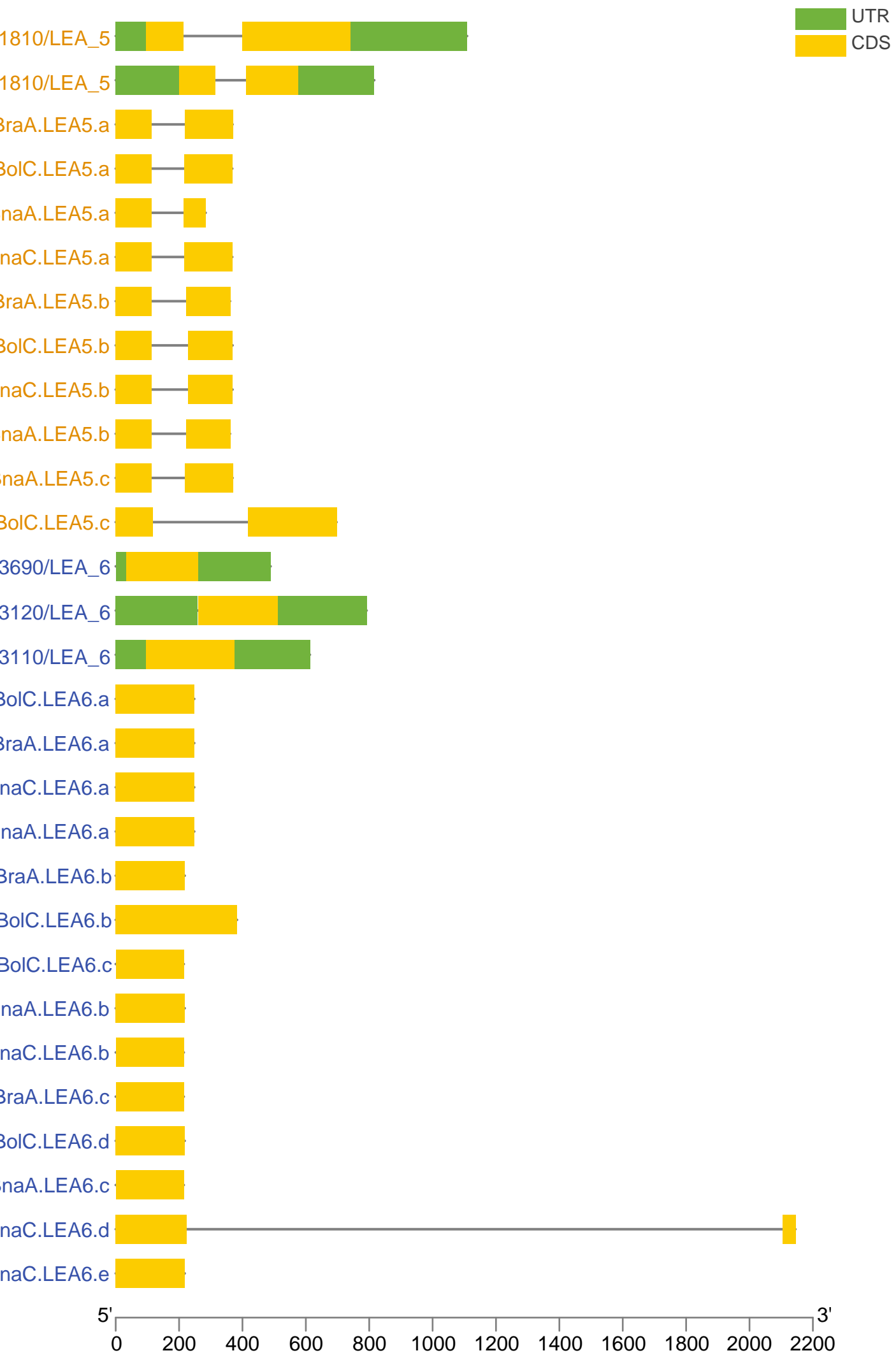

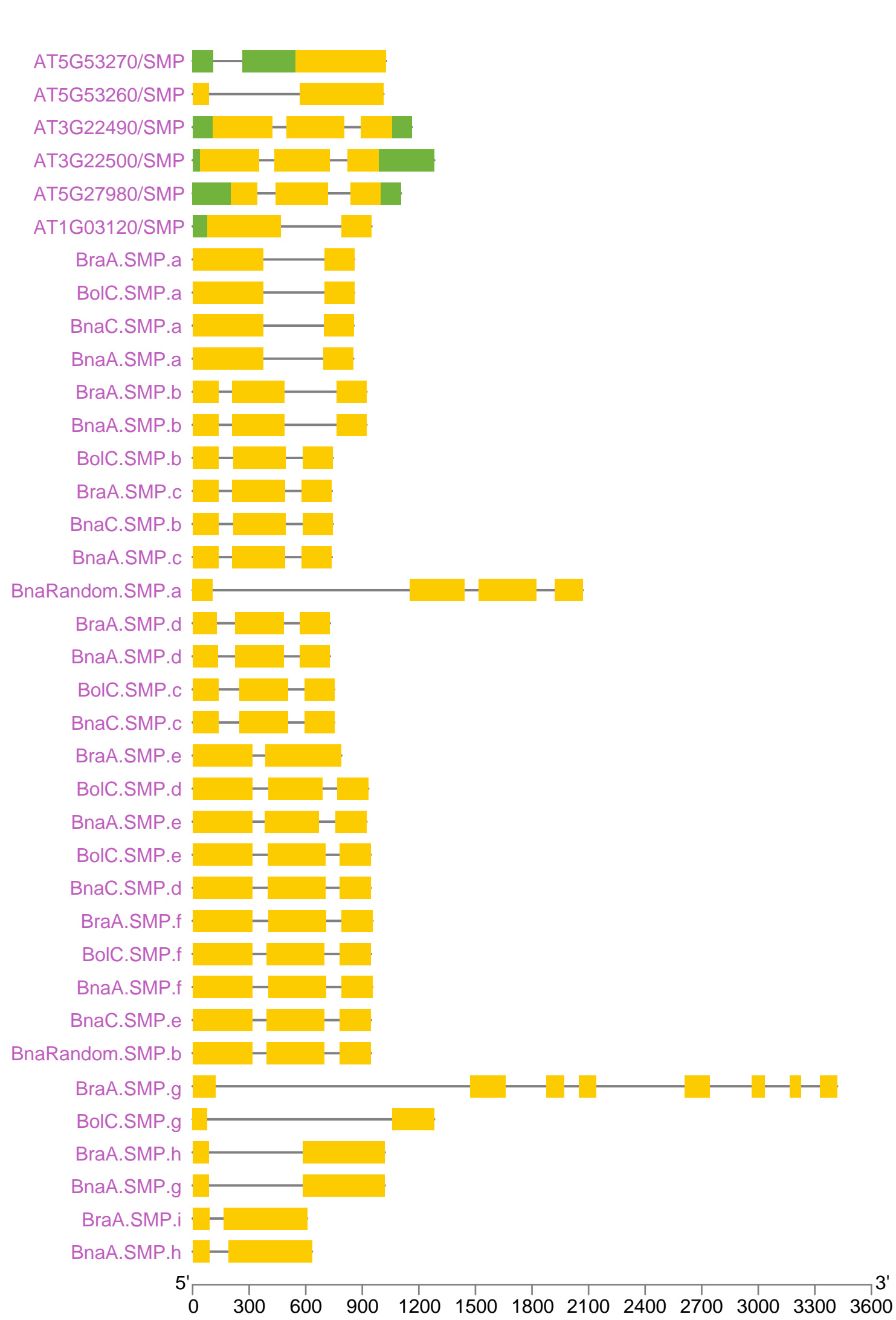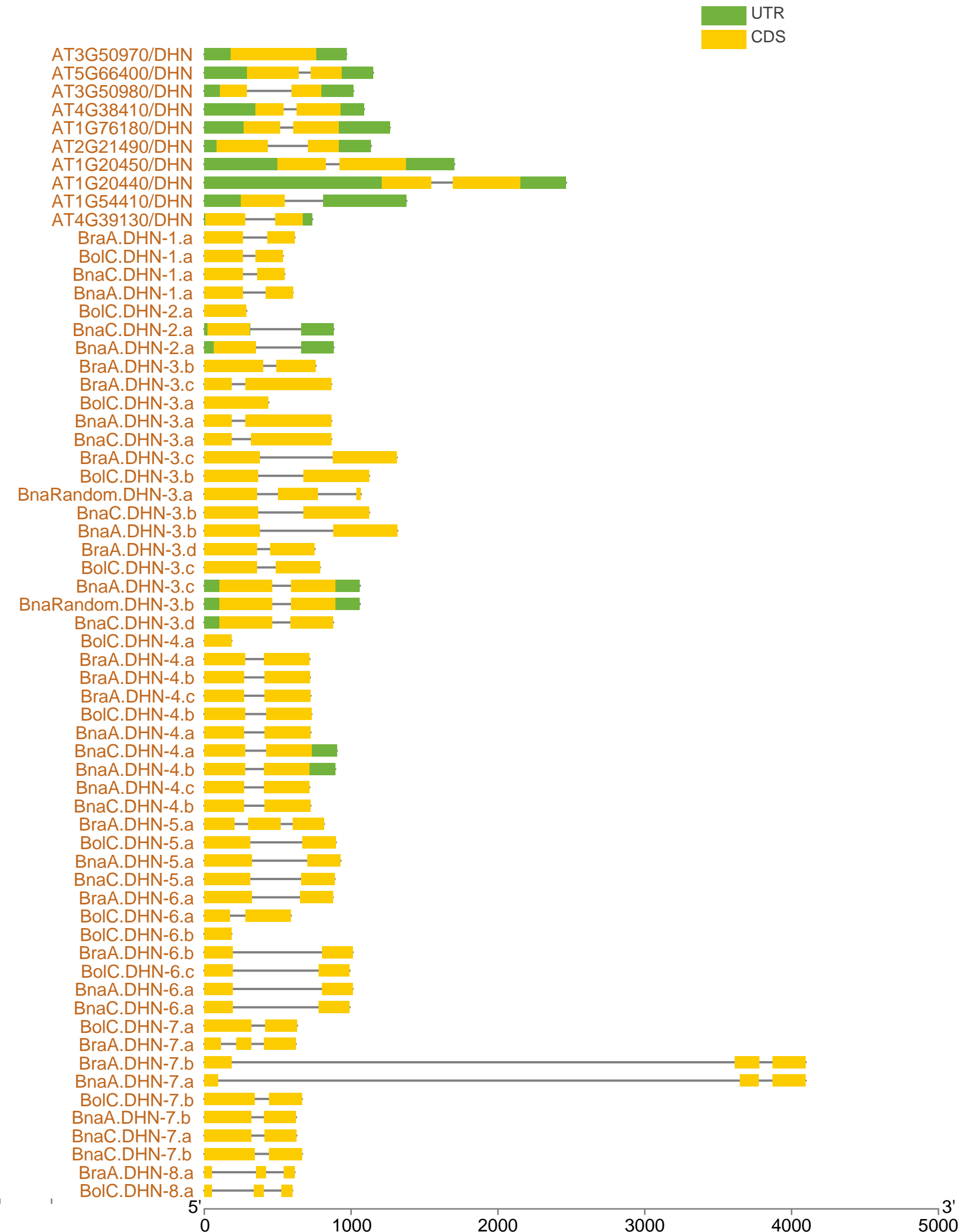

Supplement: Supplementary file 6 — Supplementary Material 6: Supplementary Figure 2. Gene structures of LEA genes analyzed by TBtools. Green boxes, black lines, and orange boxes indicate untranslated regions, introns, CDS, respectively. [file 12870_2024_5111_MOESM6_ESM.pdf]

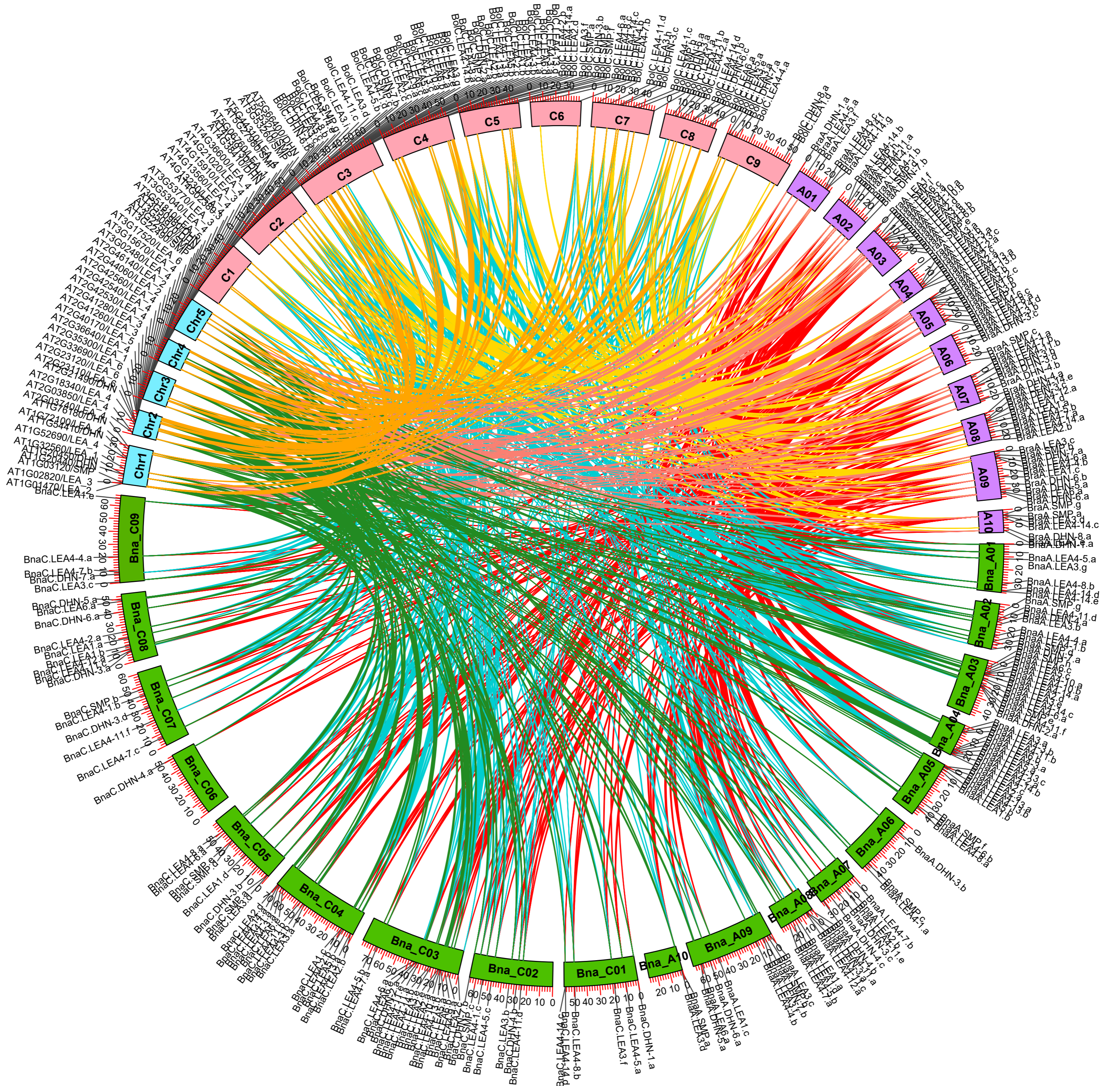

Supplement: Supplementary file 7 — Supplementary Material 7: Supplementary Figure 3. Syntenic relationships among LEA genes of B. napus, B. rapa, B. oleracea and Arabidopsis. The chromosomes of B. napus, B. rapa, B. oleracea and Arabidopsis were shown in green, purple, and pink, and blue respectively. The Bna_Random, A0_Random and C0_Random chromosome fragment is very short and is not marked in the figure. The orthologous and paralogous LEA genes were mapped onto the chromosomes/scaffolds and linked with each other. The syntenic LEA gene pairs from B. rapa and Arabidopsis, B. oleracea and Arabidopsis, B. napus and Arabidopsis, B. rapa and B. oleracea, B. rapa and B. napus, B. oleracea and B. napus, were linked by pink, orange, green, gold, red, and blue lines, respectively. [file 12870_2024_5111_MOESM7_ESM.pdf]

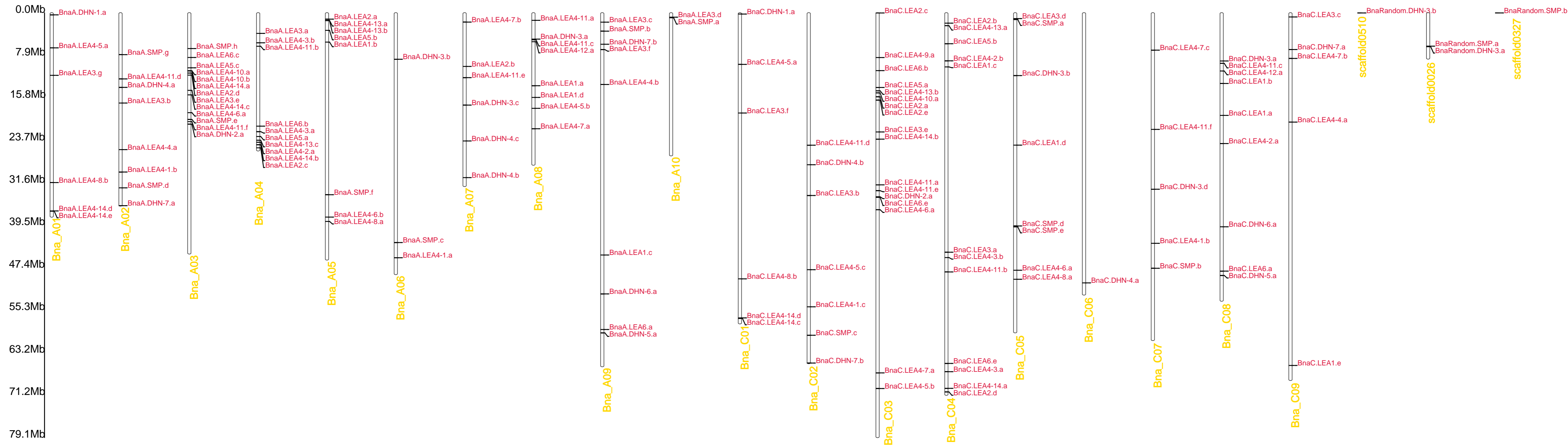

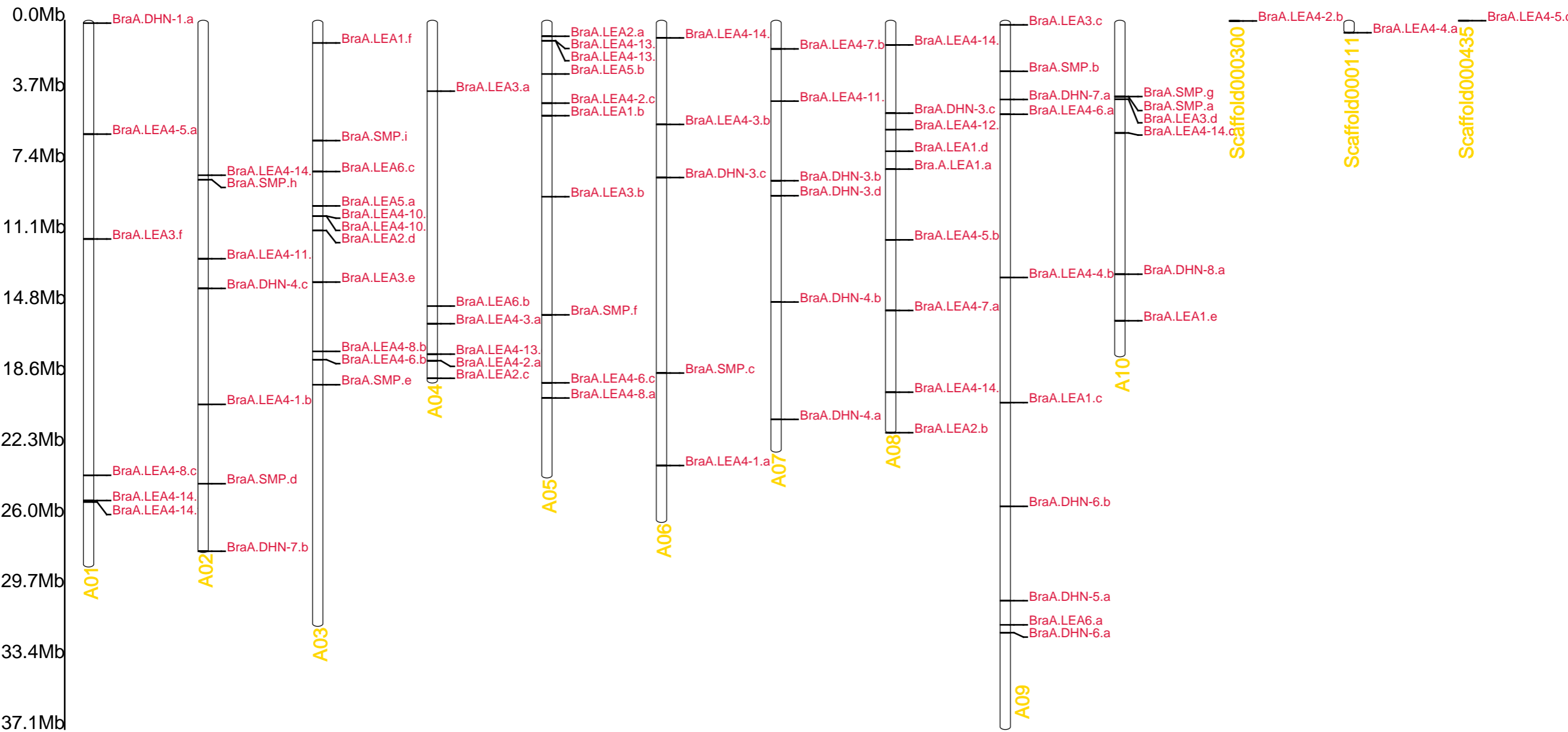

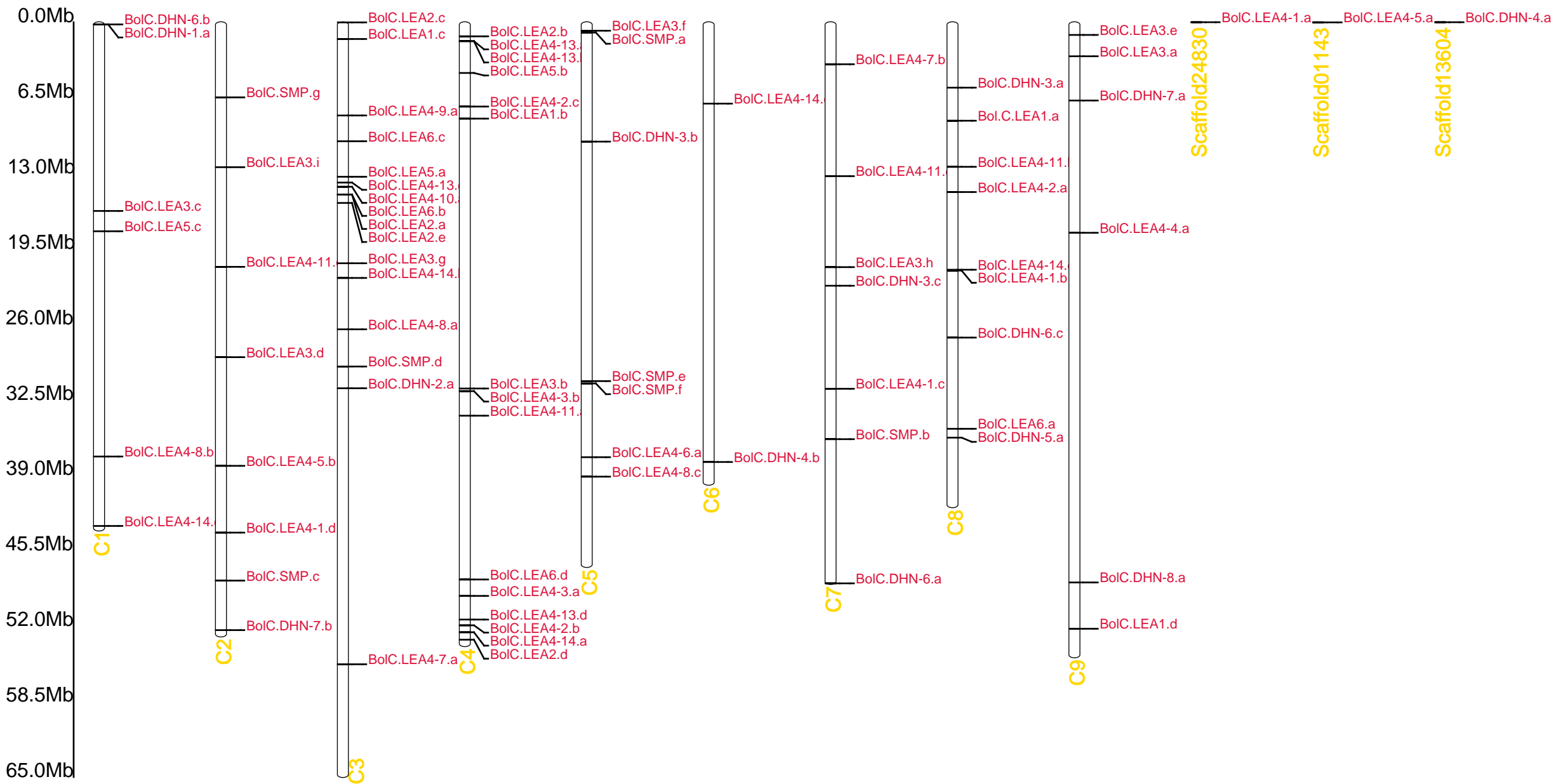

Supplement: Supplementary file 8 — Supplementary Material 8: Supplementary Figure 4. Distribution of LEA genes on the B. rapa (A), B. oleracea (B) and B. napus (C) chromosomes. (A) A01–10: B. rapa chromosomes, Scaffold000300, Scaffold000111 and Scaffold000435: unanchored scaffolds from B. rapa; (B) C1–9: B. oleracea chromosomes, Scaffold24830, Scaffold01143 and Scaffold13604: unanchored scaffolds from B. oleracea; (C) Bna_A01–10: B. napus An-subgenome chromosomes; Bna_C01–09: B. napus Cn-subgenome chromosomes; scaffold0510, scaffold0026 and scaffold0327: unanchored scaffolds from B. napus. [file 12870_2024_5111_MOESM8_ESM.pdf]

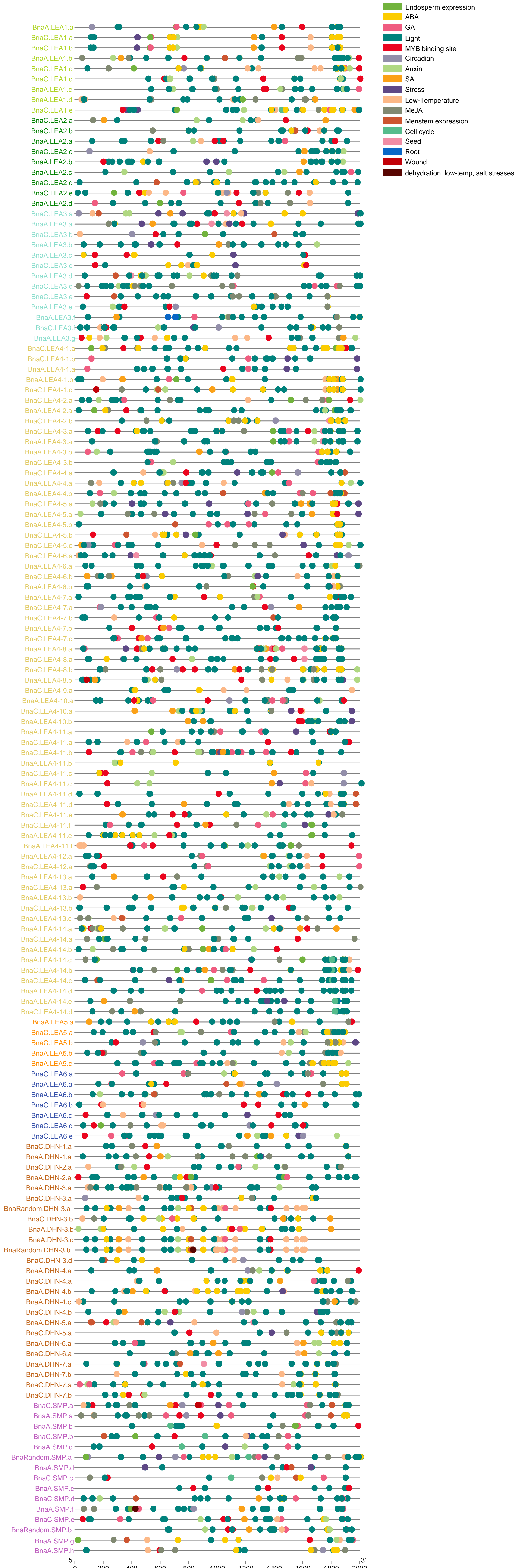

Supplement: Supplementary file 9 — Supplementary Material 9: Supplementary Figure 5. The cis-regulatory elements in the promoters of BnaLEAs predicted by PlantCARE. Boxes filled with different colors represent different cis-regulatory elements. [file 12870_2024_5111_MOESM9_ESM.pdf]

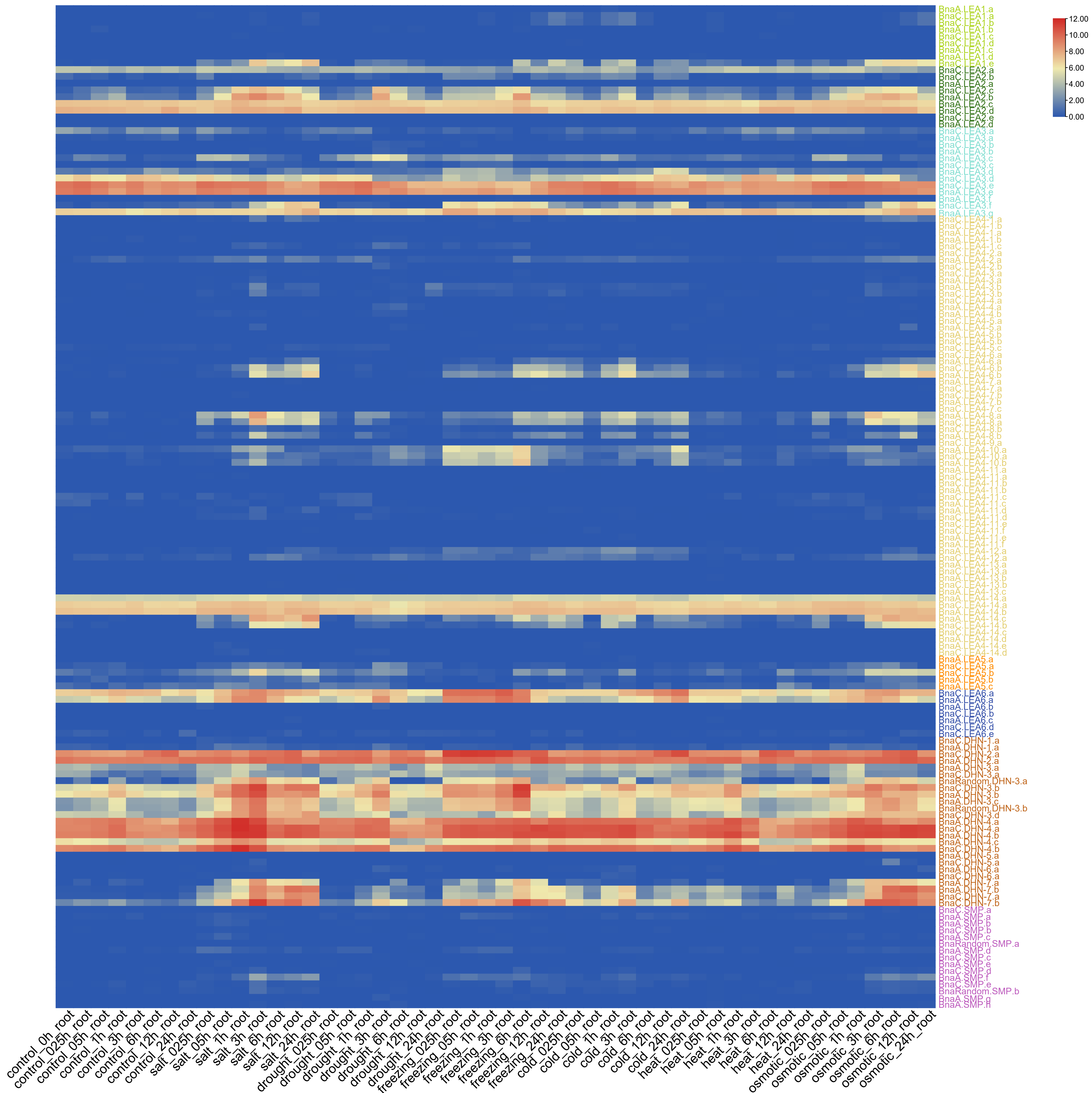

Supplement: Supplementary file 10 — Supplementary Material 10: Supplementary Figure 6. Expression of BnaLEA genes under different abiotic stresses in roots. Colored rectangles indicate expression levels of BnaLEA genes. Red means high expression, Blue means low expression. Color from red to blue represents descending log2(TPM+1). [file 12870_2024_5111_MOESM10_ESM.pdf]

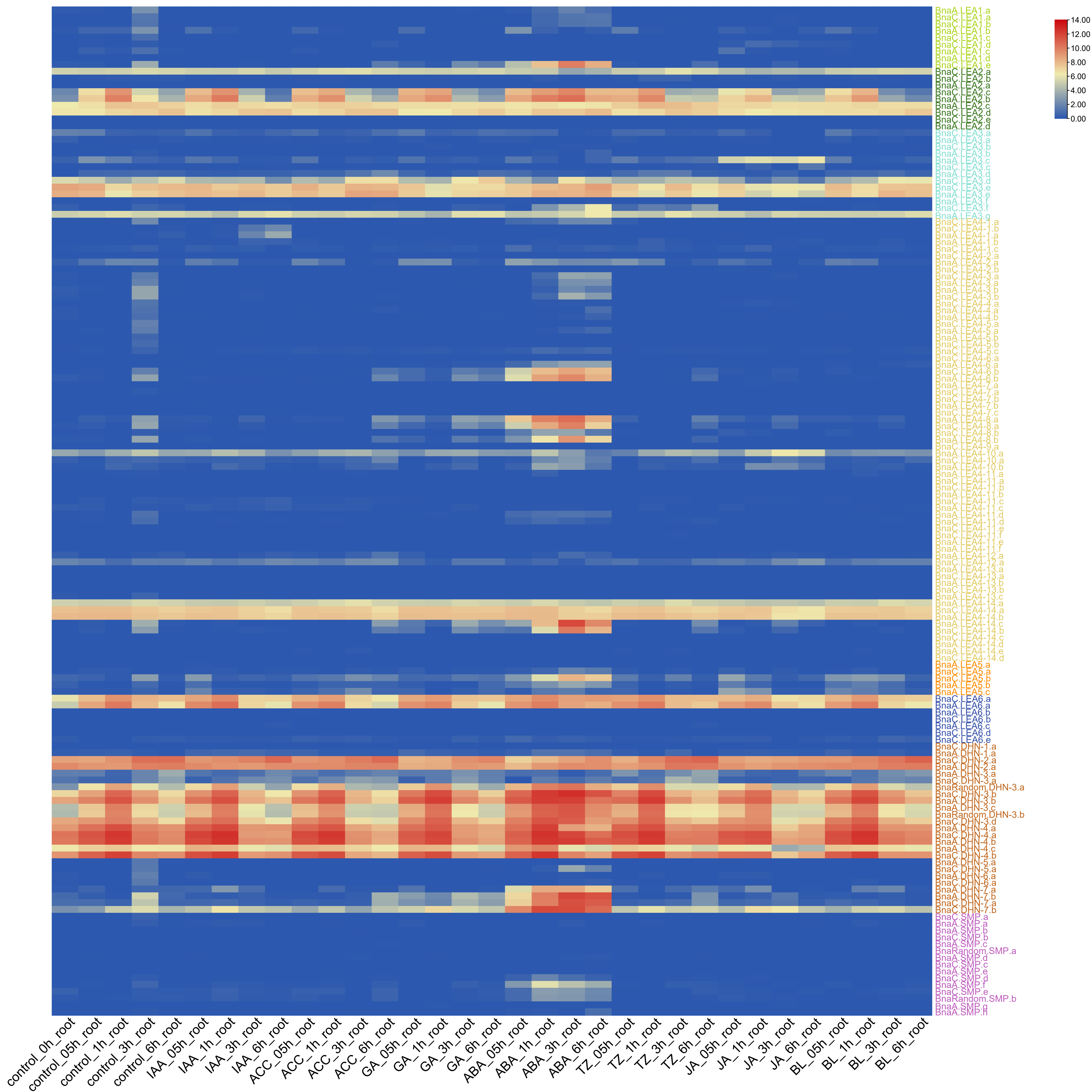

Supplement: Supplementary file 11 — Supplementary Material 11: Supplementary Figure 7. Expression of BnaLEA genes under hormones treatments in roots. Colored rectangles indicate expression levels of BnaLEA genes. Red means high expression, Blue means low expression. Color from red to blue represents descending log2(TPM+1). [file 12870_2024_5111_MOESM11_ESM.pdf]
